# Symbiont‐Induced HSP83 Establishes Two‐Tiered Control of Antifungal Immunity in an Invasive Beetle–Fungus Complex

**DOI:** 10.1002/advs.77421

**Published:** 2026-08-27

**Authors:** Qingtai Yang, Zhudong Liu, Yuqiao Yin, Jing Su, Dunyang Yu, Longsheng Xing, Zhen Zou, Le Kang, Jianghua Sun

**Affiliations:** ^1^ Hebei Basic Science Center For Biotic Interactions School of Life Sciences Institutes of Life Science and Green Development Hebei University Baoding China; ^2^ State Key Laboratory of Animal Biodiversity Conservation and Integrated Pest Management Institute of Zoology Chinese Academy of Sciences Beijing China

**Keywords:** antifungal immunity, biological invasion, heat shock proteins, host‐microbe interactions, immune modulation, symbiont‐mediated adaptation, symbiosis

## Abstract

Hosts must distinguish mutualistic symbionts from antagonists while avoiding harmful immune overactivation, yet the mechanisms maintaining this balance remain unclear. This challenge is acute during biological invasions, where exposure to novel microbial communities can outpace host genetic adaptation. *Dendroctonus valens* (RTB) associates with its mutualistic fungus *Leptographium procerum* (Lp), forming an invasive beetle–fungus complex during attacks on Chinese pines. Lp fails to trigger antimicrobial peptide expression but induces the heat shock protein 83 (HSP83). The native antagonist *Ophiostoma minus* (Om) activates immunity through pattern recognition receptors, including PGRP‐SA, PGRP‐SC2, βGRP3, and βGRP5. HSP83 modulates these responses through two‐tiered negative regulation. First, it associates with PGRP‐SA, βGRP3, and βGRP5 to attenuate Om detection. Second, it interacts with NF‐κB–like factor Dorsal to limit antimicrobial peptide production. This symbiont‐induced regulation enables selective defense, supporting RTB survival during combined Lp and Om exposure while limiting excessive Toll‐dependent PRR/AMP activation and easing immune–metabolic trade‐offs. These findings define a symbiont‐assisted, mutation‐independent mechanism of host adjustment during biological invasions, in which microbial partners provide immediate immune benefits and influence host success in novel environments.

## Graphical abstract

In an invasive beetle–fungus complex, the mutualist *Leptographium procerum* induces host HSP83 and modulates Toll‐dependent antifungal immunity. HSP83 mediates two‐tiered control by associating with selected fungal recognition receptors and restraining Dorsal‐driven antimicrobial peptide transcription. This symbiont‐mediated immune regulation limits excessive responses to the native antagonist *Ophiostoma minus* while supporting host survival during invasion.

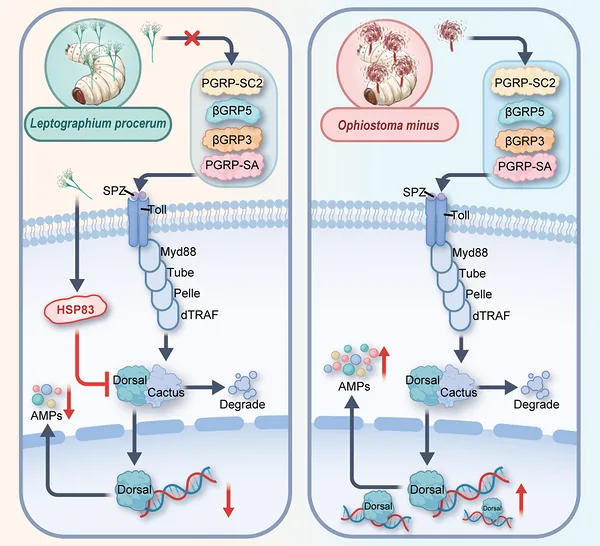

## 1 Introduction

Maintaining symbiotic relationships with microbial communities requires hosts to mount distinct immune responses to pathogens versus commensals [1]. The underlying mechanisms have been most clearly defined in selected model systems across animals and plants [2, 3, 4]. In humans, the gut metabolite butyrate induces bactericidal antimicrobial peptides and limits mucosal exposure to luminal microbes, thereby curbing aberrant inflammation [2, 3]. In *Arabidopsis thaliana*, the bacterial elicitor flg22 activates root immune signaling. This response antagonizes IMA1‐dependent iron‐deficiency pathways and limits iron availability to potential pathogens [4]. However, most mechanistic insight derives from bacterial systems, and functional studies on commensal fungi remain relatively scarce. Although recent work has delineated fungal immune evasion and pathogenic strategies in several hosts, the molecular basis by which hosts regulate immune responses to diverse fungal communities remains poorly understood.

The immune system is central to maintaining equilibrium between hosts and their commensal microorganisms [5]. Insects interact with diverse fungal partners, ranging from mutualists to antagonists, and rely on evolutionarily conserved innate immune signaling pathways, making them tractable models to dissect context‐dependent innate immune regulation in vivo. Antimicrobial peptides (AMP) constitute a major humoral defense against microbes in insects [6]. AMP transcription is primarily controlled by the Toll and immune deficiency (IMD) pathways [7], which are activated when pattern‐recognition receptors (PRRs) such as β‐1,3‐glucan recognition proteins (βGRPs) and peptidoglycan recognition proteins (PGRPs) detect pathogen‐associated molecular patterns (PAMPs) on microbial surfaces [8]. In the IMD pathway, PGRP‐LC and PGRP‐LE sense Gram‐negative bacteria, whereas Toll signaling depends on PRRs including PGRP‐SA and βGRPs to recognize Gram‐positive bacteria and fungi [9]. Upon activation, the NF‐κB–like transcription factors Dorsal and Relish enter the nucleus and induce AMP expression, promoting host protection [10]. Although the core components of innate immunity have been well characterized in many insects [9, 10, 11], recent evidence suggests that orthologs of mammalian immune regulators also modulate insect immune signaling. For example, mosquito OTU7B inhibits Toll signaling by blocking Rel1 nuclear entry [12], and heat shock proteins (HSP90, HSP70, HSP60, and HSP27) modulate antifungal responses in *Galleria mellonella* [13]. However, inhibitors that fine‐tune antifungal immunity and sustain fungal community balance remain poorly defined.

The invasive red turpentine beetle (*Dendroctonus valens* LeConte, RTB) and its mutualistic fungus *Leptographium procerum* (Lp) form a destructive invasive complex in China. Since its introduction from North America, this complex has killed more than ten million healthy *Pinus tabuliformis* trees [14]. Beetle–microbe interactions contribute to invasion success and are shaped by climatic and biotic drivers acting across scales, with symbiont‐mediated effects on insect invasions now well recognized [15, 16]. Conifer terpene volatiles function as semiochemicals that structure host defense and mediate beetle attraction, providing context for the volatile cues observed in this system [17]. In China, a distinct Lp genotype with enhanced pathogenicity toward native pines has been identified, and this symbiont further induces host plants to release higher amounts of 3‐carene, a primary attractant for RTB adults [18]. RTB‐associated bacteria further contribute to invasion by degrading pine defensive phenolics such as naringenin [19], and alleviating nutritional competition between beetle larvae and Lp [20]. However, invasive species inevitably encounter native antagonists in invaded habitats. In the RTB system, invasion is constrained by native fungi, most notably the antagonist *Ophiostoma minus* (Om) [21, 22]. Om is known to impair larval development in multiple *Dendroctonus* species through antagonistic interactions with mutualistic fungi [23]. Om suppresses larval feeding and growth, and is associated with elevated phenoloxidase activity, although a direct causal reduction in host fitness via a defined prophenoloxidase cascade has not been demonstrated [21, 22]. Despite extensive studies on RTB–fungus associations, the molecular mechanisms that enable concurrent tolerance of the mutualist Lp and defense against the native antagonist Om remain unknown.

In this study, we used the invasive beetle RTB, which naturally associates with the mutualist Lp and encounters the antagonist Om, to dissect context‐dependent antifungal immune regulation. We show that Lp induces heat shock protein 83 (HSP83), which attenuates Om‐triggered immune responses. HSP83 regulates Toll‐related antifungal immunity at two sequential tiers, attenuating PRR‐mediated Om recognition and restraining Dorsal‐dependent antimicrobial effector transcription. Together, these findings reveal a symbiont‐induced chaperone that mediates context‐dependent antifungal immune regulation during exposure to mutualistic and antagonistic fungi.

## 2 Results

### 2.1 *L. procerum*‐Mediated Tolerance to *O. minus* in RTB

**FIGURE 1:**
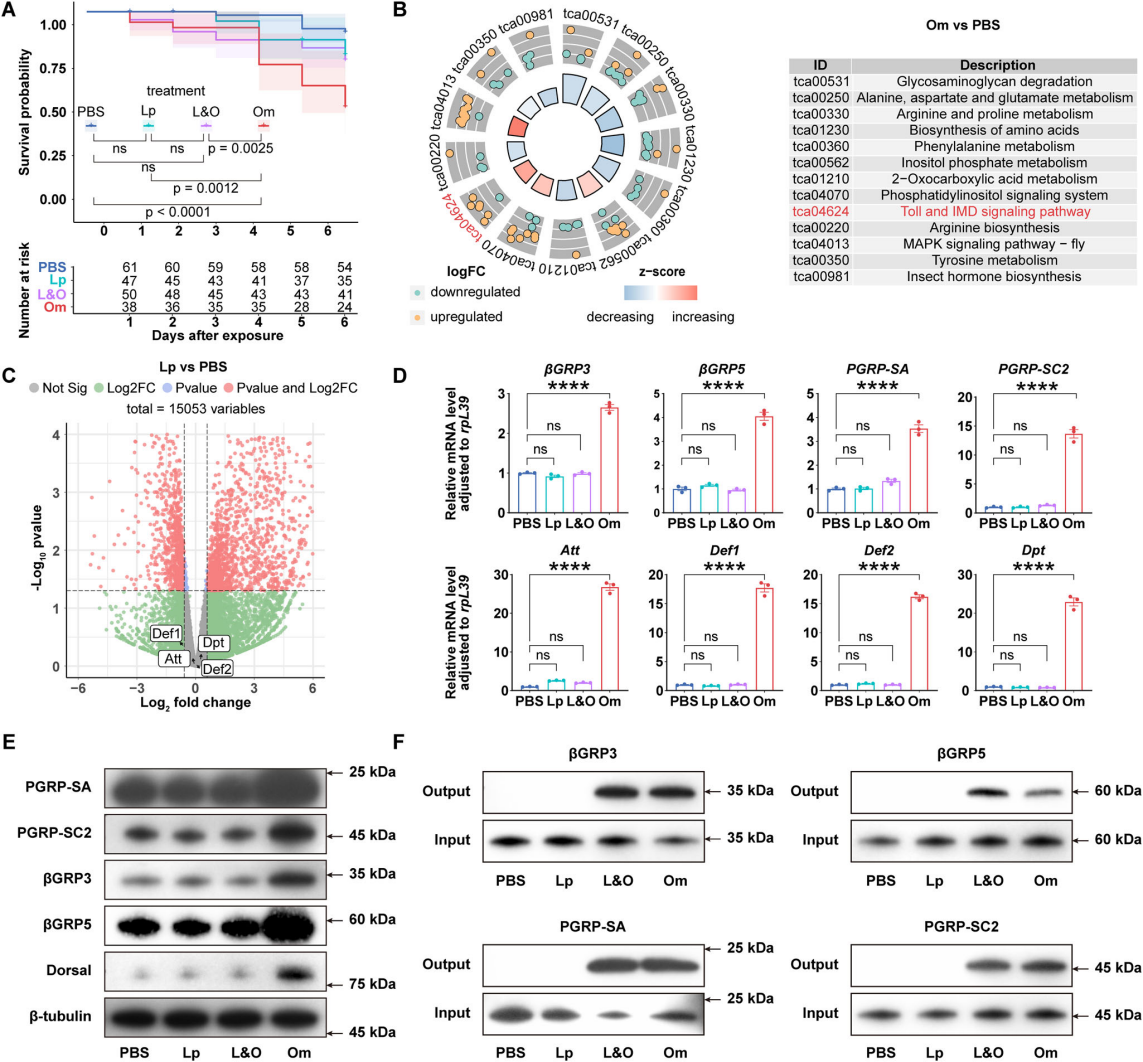
*L. procerum* helps RTB overcome *O. minus* lethal effects and attenuates antifungal immune activation. (A) Survival curves of surface‐sterilized third‐instar RTB larvae exposed to Lp, L&O, Om, or PBS control. Combined Lp and Om exposure partially rescued Om‐induced mortality. *n* ≥ 38 per group. Log‐rank test. (B) KEGG enrichment of 804 differentially expressed genes (DEGs; fold change > 1.5 or < 0.67, *p* < 0.05) from Om vs. PBS transcriptomes. PBS served as the reference condition. Circle plot shows 13 significantly enriched pathways (64 DEGs total), highlighting immune signaling pathways. (C) Volcano plot of 15,053 genes in Lp vs. PBS transcriptomes. PBS served as the reference condition. Antimicrobial peptide (AMP) genes (*Attacin*, *Defensin1/2*, *Diptericin*) showed no significant regulation. Significance threshold: fold change > 1.5 or < 0.67, *p* < 0.05 (two‐sided *t*‐test). (D) qRT‐PCR validation of PRR and AMP expression at 96 h after conidial exposure. Om significantly induced both PRR and AMP transcription, whereas Lp and L&O did not. *rpL39* used for normalization. Mean ± SEM, *n* = 3 biological replicates. One‐way ANOVA with Dunnett's test. ns, not significant; **** *p* < 0.0001. (E) Western blot analysis of PRRs (PGRP‐SA, PGRP‐SC2, βGRP3, βGRP5) and Dorsal at 96 h after conidial exposure. Protein levels were elevated in Om exposure and reduced in L&O treatments. *n* = 3 larvae/treatment. β‐tubulin as loading control. (F) In vitro binding assays of recombinant PRRs with fungal spores. Bound fractions detected with anti‐V5 antibody. Strong PRR binding was observed for Om and L&O, whereas no binding was detected for Lp. Original, unaltered full blot images corresponding to panels (E) and (F) are provided in Figure S6.

To investigate whether the two fungal partners elicited distinct immune responses, surface‐sterilized third‐instar RTB larvae were exposed by immersion to conidial suspensions of Lp, Om, a mixture of Lp and Om (L&O), or PBS as controls, and daily survival was monitored. Compared with the PBS group, Om exposure markedly reduced larval survival (Figure 1A). Combined exposure to Lp and Om partially rescued Om‐induced mortality, whereas Lp exposure or L&O treatment had minimal impact on survival relative to PBS (Figure 1A).

To elucidate the underlying mechanism, RNA sequencing (RNA‐seq) was performed at 96 h after conidial exposure. In Om‐exposed samples, 804 genes were differentially expressed (fold change > 1.5 or < 0.67, *p* < 0.05). KEGG pathway analysis revealed significant upregulation of Toll and IMD signaling genes and concurrent downregulation of glycosaminoglycan degradation genes (*p* < 0.05; Figure 1B). A heatmap analysis showed that Om exposure induced multiple Toll pathway components (*Dorsal*, *Toll3*, *Spz4*, *MyD88*, *Tube*, *Pelle*, *TRAF6*; Figure S1A), whereas Lp and L&O treatments increased expression of inhibitory serpins (*Serpin3*, *Serpin9*, *Serpin10*; Figure S1A). Considering that the Toll and IMD pathways regulate AMP production in insects [7], we analyzed AMP expression across 15,053 detected genes. A volcano plot comparison revealed no significant AMP changes in Lp versus PBS samples (Figure 1C).

The Toll signaling pathway is a primary antifungal defense in insects and detects microbes through PRRs [10]. We next quantified mRNA levels of upstream PRRs within the Toll cascade. The four receptors *PGRP‐SA*, *PGRP‐SC2*, *βGRP3*, and *βGRP5* were markedly induced in Om‐exposed larvae relative to Lp, L&O, and PBS controls (Figure 1D). The remaining PRRs (*βGRP1*, *βGRP2*, *βGRP4*, *PGRP‐SC1*) showed no significant change (Figure S1B). AMP transcript patterns mirrored PRR expression, consistent with immune activation in Om‐exposed RTB (Figure 1D). AMP transcript levels were lower in the L&O group than in Om alone (Figure 1D). Western blot analysis at 96 h after conidial exposure corroborated these results. PGRP‐SA, PGRP‐SC2, βGRP3, and βGRP5 proteins accumulated to higher levels in Om‐exposed larvae but were reduced in Lp, L&O, and PBS treatments (Figure 1E). The NF‐κB–like factor Dorsal also exhibited increased abundance after Om exposure but lower expression in the L&O treatment (Figure 1E).

To assess whether PRRs discriminate between fungal partners, recombinant DvPGRP‐SA, DvPGRP‐SC2, DvβGRP3, and DvβGRP5 were incubated with Lp, L&O, or Om spores (PBS as control). Immunoblotting detected strong PRR‐binding signals for Om and L&O, whereas no binding was detected for Lp (Figure 1F). The binding patterns showed that these PRRs interacted with Om spores but not with Lp. L&O spores were bound by all four PRRs but did not elicit the marked Toll‐related immune activation observed with Om alone (Figure 1D,E). Given this observation, we examined whether Lp exposure alters Toll pathway inhibitors that might contribute to this protection. Analysis of *Serpin3*, *Serpin9*, and *Serpin10* transcripts revealed mild elevation in Lp and L&O groups (Figure S1A) without significant differences among treatments (Figure S1C), suggesting that other inhibitory factors may contribute to the observed protection.

### 2.2 HSP83 Induction by *L. procerum* Supports Host Survival During Fungal Exposure

**FIGURE 2:**
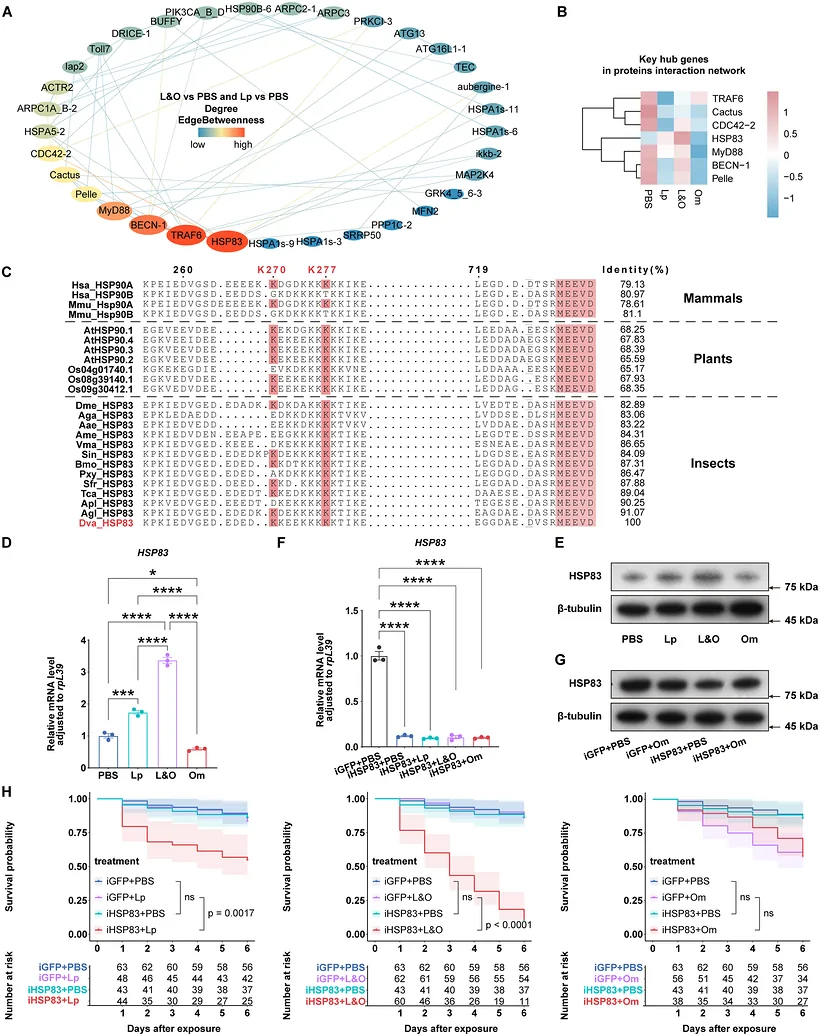
HSP83 is induced by *L. procerum* and supports RTB survival during fungal exposure. (A) PPI network comprising 33 immunity‐related DEGs showing concordant regulation in Lp‐ and L&O‐exposed larvae relative to PBS controls. Seven hub genes (red/yellow nodes) were identified by topological algorithms. Node size indicates the ClusteringCoefficient; the node fill color indicates Degree; the edge width and color indicate EdgeBetweenness. (B) Hierarchical clustering of the seven hub genes across PBS, Lp, L&O, and Om treatments at 96 h. FPKM values from three biological replicates are shown. (C) Multiple sequence alignment of DvHSP83 with HSP83 homologs from representative species, highlighting the conserved C‐terminal MEEVD pentapeptide and lysine residues K270 and K277. (D) *HSP83* expression by qRT‐PCR at 96 h after conidial exposure. Expression was significantly upregulated by Lp and L&O and reduced by Om. *rpL39* normalization. Mean ± SEM, *n* = 3. One‐way ANOVA with Dunnett's test. *p < 0.05, ***p < 0.001, ****p < 0.0001. (E) Western blot showing increased HSP83 protein in Lp‐ and L&O‐treated RTB at 96 h. *n* = 3 larvae/treatment. β‐tubulin acted as the loading control. (F) *HSP83* knockdown efficiency by qRT‐PCR at 96 h after dsRNA injection. iHSP83 significantly reduced *HSP83* transcript levels across all treatments. iGFP served as a control. *rpL39* normalization. Mean ± SEM, *n* = 3. One‐way ANOVA with Dunnett's test. ****p < 0.0001. (G) Western blot confirming HSP83 protein reduction after dsHSP83 treatment. *n* = 3 larvae/treatment. β‐tubulin acted as the loading control. (H) Survival of HSP83‐depleted (iHSP83) or control (iGFP) RTB larvae following fungal exposure. HSP83 depletion significantly reduced survival in the Lp and L&O groups. *n* ≥ 38 larvae/group. Log‐rank test. Original, unaltered full blot images corresponding to panels (E) and (G) are provided in Figure S7A,B.

Given the differential immune responses to Lp, L&O, and Om, we sought to identify potential regulatory factors mediating RTB responses to its fungal partners. Immunity‐related DEGs showing concordant regulation in Lp‐ and L&O‐exposed larvae relative to PBS controls were submitted to STRING; specifically, log_2_FC values were either greater than 0 in both comparisons or less than 0 in both comparisons. The resulting PPI network comprised 33 nodes and 54 predicted interactions and was visualized using Cytoscape (Figure 2A). Integrated topological analysis identified seven hub genes implicated in host antifungal regulation: *HSP83*, *TRAF6*, *BECN‐1*, *MyD88*, *Pelle*, *Cactus*, and *CDC42‐2*. Among these hub genes, HSP83 alone showed higher transcript abundance in the Lp and L&O groups than in the Om group and, to a lesser extent, the PBS group, whereas its abundance was lower in Om than in PBS (Figure 2B). This distinctive treatment‐associated expression pattern led us to prioritize HSP83 for further investigation.

Heat shock proteins (HSPs) comprise six major families, including HSP90, HSP70, and small HSPs [24]. Within the HSP90 family, four subtypes are recognized: cytosolic HSP90α/β, endoplasmic reticulum GRP94, and mitochondrial TRAP‐1 [25]. Although HSP90 proteins are well known for immunoregulatory roles in mammals and plants [26, 27], their functions in insect antifungal defense remain poorly understood. Phylogenetic analysis of 122 representative HSP sequences revealed that DvHSP83 clusters with insect HSP83 orthologs from Coleoptera, Lepidoptera, Diptera, and Hymenoptera, forming a distinct clade separated from other HSP90 subfamilies (Figure S2A). Conserved domain analysis confirmed the canonical HSP90 architecture with a theoretical molecular weight of 81.33 kDa (Figure S2B,C). Multiple sequence alignment showed the C‐terminal pentapeptide MEEVD, characteristic of cytosolic HSP90s [28], and lysine residues at positions 270 and 277 corresponding to the K270/K277 motif in mammalian HSP90α, previously implicated in extracellular secretion (Figure 2C) [29]. DvHSP83 therefore represents a conserved HSP90‐family protein across insects, plants, and mammals.

We validated HSP83 expression patterns by qRT‐PCR and Western blot at 96 h after conidial exposure. Both mRNA and protein levels of HSP83 were significantly elevated in Lp and L&O treatments but reduced in Om relative to PBS (Figure 2D,E). To assess the role of HSP83 in RTB responses to these fungal partners, we performed RNAi knockdown of *HSP83* followed by fungal exposure. HSP83 mRNA and protein levels were markedly decreased after dsHSP83 treatment (Figure 2F,G). Daily monitoring of larval survival showed that *HSP83* silencing significantly reduced survival in Lp‐ and L&O‐exposed groups, whereas the Om‐exposed group remained largely unaffected (Figure 2H). These results indicate that HSP83 promotes host survival under Lp and L&O challenge.

### 2.3 HSP83 Restrains Toll‐Dependent Antifungal Responses

To further define the molecular basis of this HSP83‐dependent tolerance, transcriptomic profiling was performed following HSP83 depletion. RTB larvae pretreated with dsRNA targeting *HSP83* (iHSP83) or dsEGFP (iGFP, RNAi control) were subsequently exposed to Lp, Om, L&O, or PBS, with PBS serving as the exposure control. Genes with *p* < 0.05 and a fold‐change > 2 or < 0.5 were defined as differentially expressed genes (DEGs).

**FIGURE 3:**
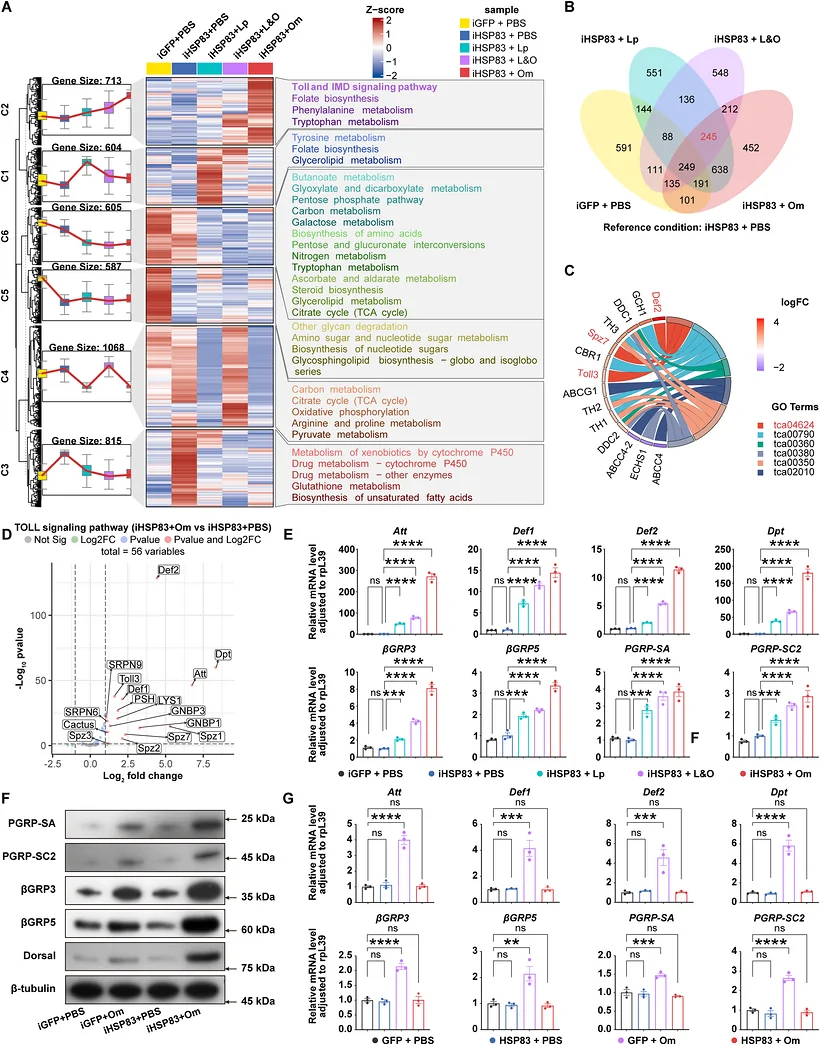
HSP83 depletion activates Toll signaling and HSP83 supplementation reduces antifungal immunity. (A) Expression trend, clustering and KEGG enrichment of 4,392 DEGs across iGFP + PBS, iHSP83 + PBS, iHSP83 + Lp, iHSP83 + L&O, and iHSP83 + Om conditions. The Toll and IMD signaling pathway was significantly enriched in the upregulated cluster C2 (*p* < 0.01). Z‐score heatmap shows mean expression (*n* = 3 replicates). (B) Venn diagram of unique and shared DEGs in iHSP83+Lp, iHSP83+L&O, iHSP83+Om, iGFP+PBS, with each group compared with the common reference iHSP83 + PBS. Red number indicates co‐regulated genes. Two‐sided *t*‐test, FC > 2 or < 0.5, *p* < 0.05. (C) KEGG enrichment of 245 co‐regulated DEGs identified in panel (B). Chord graph shows 6 pathways (*p < 0.05*) containing 14 DEGs. The Toll and IMD signaling pathway was highlighted. (D) Volcano plot of 56 Toll pathway genes in iHSP83+Om vs iHSP83+PBS. iHSP83+PBS served as the reference condition. Sixteen genes were significantly upregulated (FC > 2, *p* < 0.05). Two‐sided *t*‐test. (E) qRT‐PCR of immune genes in HSP83‐depleted RTB at 96 h after conidial exposure. Immune gene expression was elevated in HSP83‐depleted larvae following Lp, L&O, or Om exposure relative to the iGFP + PBS reference condition. *rpL39* normalization. Mean ± SEM, *n* = 3. One‐way ANOVA with Dunnett's test. ns, not significant; ***p < 0.001, ****p < 0.0001. (F) Western blot analysis of PRRs (PGRP‐SA, PGRP‐SC2, βGRP3, βGRP5) and Dorsal in iHSP83‐ and iGFP‐treated RTB at 96 h after Om exposure. All proteins accumulated after HSP83 depletion under Om exposure. *n* = 3 larvae/treatment. β‐tubulin served as the loading control. (G) qRT‐PCR showing that HSP83 protein supplementation reduces immune gene expression. RTB larvae were injected with recombinant HSP83 or EGFP and subsequently exposed to PBS or Om; transcript levels were quantified at 96 h after exposure. GFP+PBS was set to 1. *rpL39* normalization. Mean ± SEM, *n* = 3. One‐way ANOVA with Dunnett's test. ns, not significant; **p < 0.01, ***p < 0.001, ****p < 0.0001. Original, unaltered full blot images corresponding to panel (F) are provided in Figure S7C.

A heatmap analysis of all *HSP* transcripts confirmed that no other *HSPs* were affected by *HSP83* interference (Figure S3A). Analyses of expression trends and KEGG enrichment grouped the DEGs into six major clusters. Cluster C2 revealed that transcripts associated with the Toll and IMD signaling pathways were strongly increased after fungal exposure in HSP83‐depleted larvae (Figure 3A). In contrast, clusters C5 and C6 displayed significant downregulation of multiple metabolic pathways, including carbohydrate, amino‐sugar, carbon, and nitrogen metabolism (*p* < 0.05), consistent with metabolic suppression accompanying immune activation (Figure 3A).

A circular representation of pathway members further illustrated their enhanced expression after HSP83 depletion combined with fungal challenge (Figure S3B). Heatmap visualization of 93 genes from the Toll and IMD cascades showed strong upregulation of key Toll components, *Dorsal*, *Toll3*, *Spz1*, *MyD88*, *Pelle*, and *TRAF6*, in *HSP83*‐silenced larvae following fungal exposure (Figure S3C). A Venn diagram analysis of DEGs from iHSP83 + Lp, iHSP83 + L&O, iHSP83 + Om, and iGFP + PBS, all defined relative to iHSP83 + PBS, identified 245 commonly regulated genes (Figure 3B). KEGG enrichment of these genes confirmed significant representation of the Toll and IMD pathways, with notable upregulation of *Def2*, *Spz7*, and *Toll3* (Figure 3C). Volcano plot analysis indicated a stronger transcriptional response in the Toll pathway than in the IMD pathway after HSP83 depletion under Om exposure. Of the 56 Toll pathway genes and 41 IMD pathway genes examined, 16 and 3 were significantly upregulated, respectively (Figure 3D; Figure S3D).

We next systematically examined AMPs and PRRs that were upregulated after Om exposure in HSP83‐depleted larvae. qRT‐PCR analysis showed that *PGRP‐SA*, *PGRP‐SC2*, *βGRP3*, *βGRP5*, and AMP genes (*Att*, *Def1*, *Def2*, *Dpt*) were all elevated in HSP83‐depleted larvae following fungal exposure relative to the iGFP + PBS reference condition (Figure 3E). Likewise, *HSP83* RNAi markedly enhanced expression of *PGRP‐SA*, *PGRP‐SC2*, *βGRP3*, *βGRP5*, and *Dorsal* after Om exposure compared with the iGFP + Om treatment (Figure 3F). No significant changes were observed under PBS treatment following HSP83 depletion, indicating that HSP83 primarily restrains Om‐induced accumulation of these immune proteins rather than their basal abundance (Figure 3F). Conversely, injection of recombinant HSP83 protein markedly reduced expression of AMP and PRR genes (*Att*, *Def1*, *Def2*, *Dpt*, *PGRP‐SA*, *PGRP‐SC2*, *βGRP3*, *βGRP5*) following Om exposure (Figure 3G). Together, these results demonstrate that HSP83 restrains Toll‐mediated antifungal immunity in RTB and limits excessive immune activation during fungal challenge.

### 2.4 HSP83 Association With Selected PRRs Attenuates *O. minus* Recognition

**FIGURE 4:**
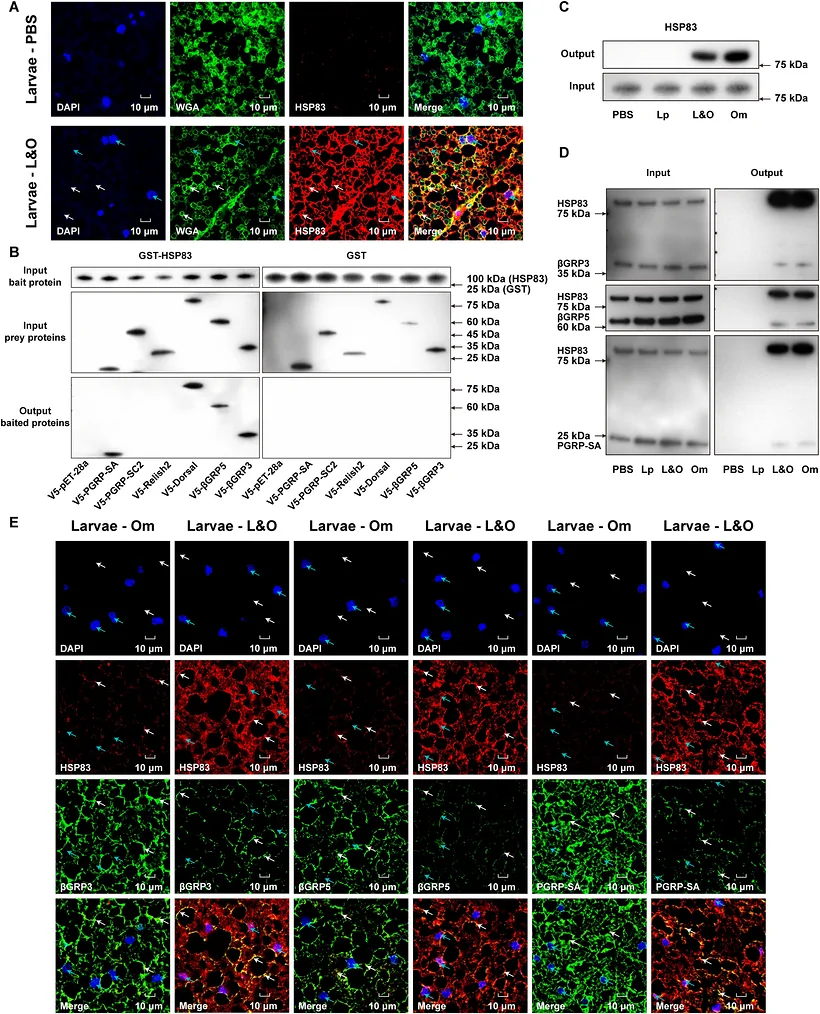
HSP83 associates with selected PRRs and attenuates *O. minus* recognition. (A,E) Immunofluorescence showing HSP83 localization relative to the WGA‐labeled plasma membrane boundary in PBS‐ and L&O‐exposed larvae (A), and spatial overlap between HSP83 and PGRP‐SA, βGRP3, or βGRP5 in Om‐ and L&O‐exposed larvae (E), at 96 h. HSP83 signals overlapped the plasma membrane boundary (white triangles) and were also detected in the cytoplasm and nucleus (blue triangles). Enhanced spatial overlap between HSP83 and PRR signals along the plasma membrane boundary was observed in the L&O treatment. DAPI (blue) marks nuclei. Scale bar: 10 µm. (B) GST pull‐down confirmed direct HSP83 interactions with PGRP‐SA, βGRP3, βGRP5, and Dorsal, but not with PGRP‐SC2 or Relish2. Anti‐GST detects bait proteins; anti‐V5 detects prey proteins. (C) In vitro binding assays of recombinant HSP83 with fungal spores. (D) In the presence of DvHSP83, recovery of PGRP‐SA, βGRP3, and βGRP5 was reduced in Om‐ and L&O‐bound fractions. Bound fractions detected by anti‐V5 antibody. Original, unaltered full blot images corresponding to panel (B) and panels (C–D) are provided in Figures S8 and S9, respectively, in the Supporting Information.

Although HSP90 proteins are generally intracellular chaperones [30], evidence from mammals, plants, and insects suggests that some members can localize extracellularly and engage immune receptors [26, 27, 31]. Given our observation that HSP83 regulates PRR expression (Figures 3F,G), we hypothesized that HSP83 may directly interact with PRRs in RTB. To examine this possibility, we performed immunofluorescence analysis using Wheat Germ Agglutinin (WGA) to visualize the plasma membrane. Immunofluorescence analysis revealed enhanced HSP83 signals overlapping the plasma membrane boundary (Figure 4A; Figure S4A, filled white triangles), with concurrent cytoplasmic and nuclear signals, a pattern consistent with pericellular association rather than purely cytoplasmic confinement (Figure 4A; Figure S4A, filled blue triangles). This pattern suggests partial membrane association rather than classical secretion.

To determine whether membrane‐associated HSP83 interacts with immune receptors, GST pull‐down assays were performed. These assays confirmed direct interactions of HSP83 with PRRs (PGRP‐SA, βGRP3, βGRP5) and with the transcription factor Dorsal (Figure 4B), suggesting that HSP83 engages both upstream recognition and downstream signaling components.

Because PRRs mediate microbial surveillance through PAMP recognition, we examined whether HSP83 association influences their fungal recognition capacity. Immunoblot assays showed that recombinant DvHSP83 bound L&O and Om spores but not Lp, resembling PRR binding profiles (Figure 4C). When pre‐mixed with PRRs (PGRP‐SA, βGRP3, βGRP5), DvHSP83 reduced PRR recovery in Om‐bound fractions, supporting interference with PRR–fungus association in these in vitro assays (Figure 4D).

Modeling with AlphaFold Server was used to prioritize E15, E61, and K64 for alanine substitution analysis. Variants with E15A showed no detectable association with PGRP‐SA, βGRP3, or βGRP5 and did not detectably reduce PRR recovery in fungus‐bound fractions, whereas DvHSP83‐E61A/K64A retained detectable PRR association and the capacity to reduce PRR recovery. All three variants retained detectable direct binding to L&O and Om conidia but not to Lp conidia; together, these results identify E15 as a functionally important determinant of DvHSP83–PRR association and indicate that E61/K64 are not required for detectable association under the conditions tested (Figure S4B–D and Table S2).

To assess the spatial association of HSP83 with these PRRs in RTB tissues, immunofluorescence analysis was performed on RTB larvae challenged with L&O or Om. In L&O‐exposed larvae, fluorescent signals of PGRP‐SA, βGRP3, and βGRP5 were reduced compared with Om, while HSP83 upregulation produced distinct overlapping signals with these PRRs along the plasma‐membrane boundary (Figure 4E; Figure S4E, filled white triangles). This pattern is consistent with a spatial association between HSP83 and these PRRs during L&O challenge. Together, these findings support a working model in which DvHSP83 associates with PRRs and interferes with PRR‐mediated Om recognition, although the precise biochemical mode remains unresolved.

### 2.5 HSP83 Restrains Dorsal‐Dependent Transcription

**FIGURE 5:**
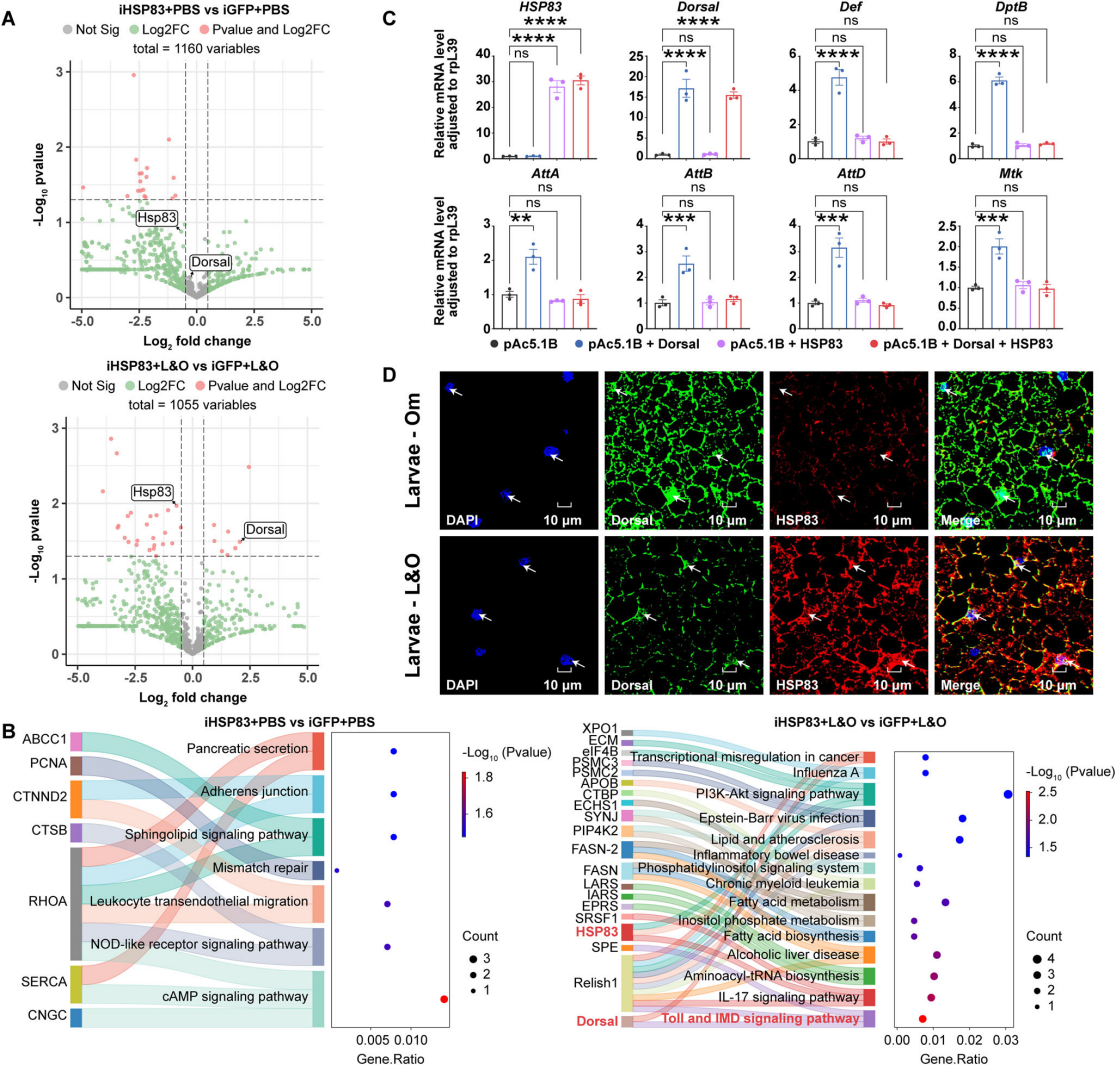
HSP83 constrains Dorsal‐dependent transcription in the Toll pathway. (A) Volcano plots showing differential protein abundance within anti‐HSP83 Co‐IP fractions for iHSP83+PBS vs iGFP+PBS (top) and iHSP83+L&O vs iGFP+L&O (bottom). In each comparison, the second group served as the reference condition. HSP83 and Dorsal were highlighted (black triangles). FC cutoffs: > 1.4 or < 0.71, *p* < 0.05. Two‐sided *t*‐test. (B) Sankey plots linking proteins showing differential abundance in the HSP83 Co‐IP fractions to enriched KEGG pathways in iHSP83+PBS vs iGFP+PBS (left, 7 pathways and 7 proteins, iGFP+PBS served as the reference condition) and iHSP83+L&O vs iGFP+L&O (right, 15 pathways and 20 proteins, iGFP+L&O served as the reference condition). The Toll and IMD signaling pathway, HSP83, and Dorsal were highlighted in red. (C) DvHSP83 reduces AMP transcript levels induced by DvDorsal in a heterologous *Drosophila* S2 cell assay. Cells were transfected with pAc5.1b‐DvDorsal with or without pAc5.1b‐DvHSP83. AMP transcript levels were quantified by qRT‐PCR at 48 h after transfection. Empty pAc5.1b control was set to 1. *rpL32* normalization. Mean ± SEM, *n* = 3. One‐way ANOVA with Dunnett's test. ns, not significant; **p < 0.01, ***p < 0.001, ****p < 0.0001. (D) Immunofluorescence showing HSP83 (red) and Dorsal (green) co‐localization in RTB cross‐sections. Enhanced nuclear co‐localization (white triangles) in L&O vs Om treatment at 96 h. DAPI (blue) marks nuclei. Scale bar: 10 µm.

Given that HSP83 depletion enhanced Toll pathway activation under fungal challenge, we sought intracellular partners linking HSP83 to pathway control. Co‐immunoprecipitation from RTB lysates coupled with data‐independent acquisition (DIA) proteomics identified endogenous HSP83‐associated complexes isolated from mixed fat body and hemolymph. Among these, Dorsal, the principal transcription factor driving AMP expression via nuclear translocation [32], was markedly enriched in the iHSP83 + L&O vs iGFP + L&O comparison but remained unchanged in the PBS comparison (Figure 5A). Across the iHSP83 + PBS vs iGFP + PBS and iHSP83 + L&O vs iGFP + L&O groups, 1,160 and 1,055 proteins were detected, respectively. Volcano plots applying FC > 1.4 or < 0.71 and *p* < 0.05 revealed significant differences in protein abundance within the HSP83 Co‐IP fractions; notably, HSP83 protein abundance decreased in both comparisons, confirming efficient *HSP83* knockdown (Figure 5A). Parallel anti‐HSP83 Co‐IP–DIA analyses consistently detected Dorsal, with greater Dorsal recovery from Om‐exposed larvae than from L&O‐exposed larvae (Figure S5A).

We next assessed pathway‐level enrichment. KEGG analysis of proteins showing differential abundance in the HSP83 Co‐IP fractions revealed significant enrichment of the Toll and IMD signaling pathways in the iHSP83 + L&O versus iGFP + L&O comparison (*p* < 0.05). This pathway was not significantly enriched in the iHSP83 + PBS versus iGFP + PBS comparison (Figure 5B). These data place Dorsal within endogenous HSP83‐associated complexes during L&O challenge and indicate that pathway engagement depends on fungal context.

To examine whether DvHSP83 modulates transcription driven by DvDorsal in a heterologous cellular context, we co‐expressed both RTB proteins in *Drosophila* S2 cells. DvDorsal strongly induced canonical *Drosophila* AMP genes (*Def*, *DptB*, *AttA*, *AttB*, *AttD*, and *Mtk*), demonstrating its transcriptional activity in this system (Figure 5C). Co‐expression of DvHSP83 markedly reduced these AMP transcript levels, providing functional evidence that DvHSP83 can limit DvDorsal‐dependent transcription in S2 cells (Figure 5C).

Finally, we examined the spatial relationship between endogenous proteins in RTB tissues. Immunostaining revealed overlapping signals between HSP83 and Dorsal in L&O‐exposed larvae, whereas in Om‐exposed larvae HSP83 signals were minimal and Dorsal was clearly localized to hemocyte nuclei (Figure 5D; Figure S5B). Taken together with prior sections, these observations indicate that Lp‐induced HSP83 limits Dorsal‐dependent transcriptional activation during L&O challenge, thereby constraining excessive Toll pathway output.

## 3 Discussion

Using the invasive RTB–fungus system, we show that Lp actively reprograms host immunity by inducing HSP83, which mediates two‐tiered negative regulation of Toll‐related antifungal immunity. HSP83 acts at an upstream PRR‐recognition tier by associating with PGRP‐SA, βGRP3, and βGRP5 to attenuate Om recognition and at a downstream effector tier by associating with Dorsal to limit Dorsal‐dependent antimicrobial peptide transcription (Figures 4, 5). These coordinated effects attenuate PRR‐mediated recognition and Dorsal‐dependent AMP output while supporting larval survival under combined fungal exposure (Figures 1, 4 and 5). By easing immune–metabolic trade‐offs that limit invasion success, this symbiont‐driven immune regulation may provide a rapid, mutation‐independent route to adaptive advantage.

HSP83 employs two‐tiered regulation that distinguishes it from classical inhibitors acting at a single node such as A20, IRAK‐M and SOCS1 [33, 34, 35]. This two‐tiered organization reflects the ecological complexity of RTB–microbe associations. This aligns with emerging views that fungus–insect interactions operate beyond bilateral regimes, involving competitive and facilitative ties with ectomicrobiomes [36]. At the upstream recognition tier, HSP83 associates with PGRP‐SA, βGRP3, and βGRP5 and attenuates PRR‐mediated Om recognition (Figure 4) [37]. The variants containing E15A retained direct binding to L&O and Om spores but lost detectable PRR association and did not detectably reduce PRR recovery in fungus‐bound fractions. These results indicate that direct fungal binding by HSP83 alone is insufficient to account for this effect and identify E15 as a functional determinant of HSP83–PRR association under the conditions tested. Because alanine substitution may also alter local conformation, E15 should not be interpreted as an experimentally resolved atomic contact residue.

At the downstream effector tier, HSP83 associates with the NF‐κB–like factor Dorsal and limits Dorsal‐dependent antimicrobial peptide transcription when upstream recognition remains sufficient to activate signaling (Figure 5) [38]. This two‐tiered architecture resembles the organization of mammalian immune checkpoints, where early priming and later effector phases are regulated at successive points [39], yet here it represents a symbiont‐driven regulatory mode in insects. The organization may be ecologically relevant because Lp and Om are phylogenetically related and likely share overlapping PAMPs [40], raising the risk of incomplete discrimination. This additional constraint may prevent excessive AMP production without abolishing PRR‐mediated surveillance, including recognition mediated by PGRP‐SC2. PGRP‐SC2 recognizes Om but shows no detectable association with HSP83 in the pull‐down assays (Figures 1F and 4B).

The evolutionary conservation of the core Toll–Dorsal transcriptional module across insects provides a rationale for using *Drosophila* S2 cells as a heterologous platform to assess transcriptional activity driven by DvDorsal and its modulation by DvHSP83 [6, 7, 8, 9, 10, 11, 32]. However, S2 cells cannot fully reproduce the cellular environment, cofactors, regulatory network, or antimicrobial peptide repertoire of *D. valens* tissues. To our knowledge, neither Lp nor Om has been reported as a natural pathogen of *Drosophila*. Neither fungus was included in the S2 cell assay, which was designed solely to assess transcriptional activity driven by DvDorsal and its modulation by DvHSP83 rather than to model fungal infection in *Drosophila*. Accordingly, the S2 cell assay provides supportive functional evidence for the relationship between DvHSP83 and DvDorsal. It complements, rather than replaces, the evidence obtained from RTB through RNAi, supplementation with recombinant protein, endogenous protein interaction, and tissue localization experiments. DvHSP83 retains the K270/K277 lysine motif conserved in mammalian HSP90α [29], although its contribution to DvHSP83 localization or immune regulation remains to be determined.

Biological invasions provide natural experiments for studying rapid ecological and immune adjustments in host–microbe systems [41]. The phylogenetically related fungi Lp and Om co‐occur in RTB galleries of infested *P. tabuliformis* yet provoke divergent immune outcomes. Lp actively attenuates host immunity by inducing HSP83, in contrast to the passive evasion strategies reported for pathogenic fungi, showing that mutualists can proactively modulate host responses (Figures 1 and 2). Functionally, Lp resembles “probiotic” keystone species that confer colonization resistance [42, 43]. Unlike bacterial probiotics, which rely on direct antimicrobial activity [44], Lp attenuates Toll pathway output (Figure 3), a core antifungal defense conserved across the Metazoa [8]. Across systems, engineered entomopathogenic fungi can likewise deliver targeted anti‐pathogen functions, underscoring the feasibility of microbe‐mediated, rapid host protection [45]. This immune attenuation is consistent with key predictions of the enemy release hypothesis, in that it alleviates costly immune activation and is expected to enable resource reallocation toward metabolism and reproduction [46, 47], although we did not directly quantify reproductive output or invasion dynamics. These transcriptomic patterns are consistent with an immune–metabolic trade‐off. Om exposure and HSP83 depletion were each associated with upregulation of immunity‐related pathways and suppression of metabolic programs (Figures 1B and 3A). Notably, RTB achieves this adjustment through symbiont recruitment rather than host genetic change, a form of symbiont‐mediated rapid adaptation that may facilitate establishment in novel habitats.

Despite conserved structural features across kingdoms (Figure 2C; Figure S2), HSP83 diverges functionally from canonical HSP90 roles. Typically, HSP90 stabilizes client proteins through ATP‐driven cycles, thereby promoting receptor competence [48]. Examples include NLRP3 inflammasome maturation [48], HSP90–Hop/Sti1 complexes that chaperone PRR trafficking in plants [26], HSP90–SGT1–RAR1 support of *Arabidopsis* R proteins [49], and HSP90–SGT1 requirements for mammalian NLR folding [48]. In contrast to these canonical chaperoning roles, RTB HSP83 associates with selected PRRs and interferes with PRR–fungus association, thereby attenuating fungal recognition (Figure 4B–D). The precise structural interface and whether HSP83 acts through steric interference, conformational modulation, masking of fungal ligands, or formation of a multicomponent complex remain to be determined [50]. The current assays also do not establish classical competitive‐binding kinetics. Noncanonical, secreted HSP90α in mammals provides a useful parallel, as it binds LRP‐1 and enhances immune signaling [51], whereas HSP83 in RTB suppresses it. These opposing outcomes reveal remarkable plasticity at the chaperone–immunity interface and highlight how molecular cooption can refine immune discrimination without requiring receptor diversification, pointing to conserved yet functionally inverted chaperone–immune crosstalk across kingdoms.

The RTB–HSP83 system is consistent with a broader metazoan principle that recognition does not equate to elimination [52, 53]. PGRP‐SA, PGRP‐SC2, βGRP3 and βGRP5 detect both Om and Lp–Om mixtures but not Lp alone (Figure 1F). Yet only Om alone triggers AMP production, whereas Lp‐induced HSP83 suppresses effector output during combined exposure. This divergence shows that HSP83 modulates immune intensity at both the recognition and effector tiers, attenuating rather than abolishing antifungal surveillance. Similar regulatory logics sustain barrier homeostasis across metazoans. Mammalian epithelia broadly express pattern‐recognition receptors yet spatially restrict downstream responses [54], and mammalian PGLYRP1–4 act as bactericidal effectors that shape microbiota [55], whereas *Drosophila* PGRP‐LE and mammalian NOD2 primarily limit inflammation without reshaping community structure [52, 56]. These cases define a conserved post‐surveillance modulation node where tolerance and defense are balanced. HSP83 extends this principle through two‐tiered regulation, modulating PRR‐mediated recognition and downstream effector output in a context‐dependent manner. The ability to distinguish mutualistic from antagonistic fungi challenges assumptions about insect immune “simplicity.” Accumulating evidence reveals context‐dependent specificity in invertebrates. For instance, honeybees differentially deploy reactive oxygen species to manage symbionts [57], and *Drosophila* coordinates Toll and IMD pathways against fungi [58]. HSP83 thus represents a regulatory innovation, illustrating how host–symbiont interactions can generate regulatory architectures that reconcile immune tolerance with defense and extend general principles of context‐dependent immune regulation across metazoans.

Symbiont‐driven immune modulation offers conceptual links between insect and mammalian host–microbe systems, suggesting relevance to human fungal dysbiosis. In the mammalian gut, dysregulated mycobiota contributes to inflammatory bowel disease and asthma through C‐type lectin receptors and CARD9‐linked inflammasome signaling [59, 60]. Current therapies rely on broad‐spectrum antifungals that eliminate both commensals and pathogens [61]. Our finding that HSP83 enables selective tolerance to mutualists while preserving responsiveness to antagonists is consistent with the idea that precision immune modulation could serve as a potential strategy to rebalance host–mycobiota interactions by dampening excessive inflammatory outputs rather than broadly amplifying antifungal effector induction. However, our experiments were performed in a single insect system under controlled laboratory conditions, and we did not directly test therapeutic interventions or measure host fitness, disease outcomes, or invasion dynamics. The upstream cues that drive Lp‐dependent HSP83 induction also remain unknown, and additional receptors or signaling nodes beyond the Toll pathway may participate in this regulatory network. Extrapolation of these findings to mammalian disease therefore remains speculative and will require direct testing in vertebrate models.

This mechanism also reframes clinical experiences with HSP90 inhibition. Pan‐HSP90 inhibitors often fail because they disrupt essential chaperone functions [62], whereas context‐specific approaches can enhance immune therapy; for example, HSP90α‐selective inhibition augments PD‐1 blockade by reversing adaptive immune resistance [25]. Whether this conserved K270/K277 lysine motif in DvHSP83 can support isoform‐selective targeting remains to be determined [29]. Advances in targeting extracellular fungal Hsp90 suggest therapeutic opportunities for fungal sepsis and fungal dysbiosis–associated inflammation [63]. From an ecological perspective, disrupting HSP83–PRR interactions could increase RTB susceptibility to native fungal antagonists and enable pesticide‐free biocontrol strategies. Future work should define the upstream cues driving Lp‐dependent HSP83 induction, map structural determinants of selective PRR engagement, and test field predictions linking Lp abundance, HSP83 activity, and invasion success. Overall, this tripartite system exemplifies how integrating invasion biology, immunology, and evolution yields both conceptual and practical advances. It links the principle of symbiont‐mediated immune regulation to potential translational applications, from fungal community–preserving therapies to biocontrol under climate change.

## 4 Conclusion

This study identifies symbiont‐induced HSP83 as a key mediator of Toll‐dependent antifungal immunity in the invasive red turpentine beetle *Dendroctonus valens*. By engaging both upstream recognition (PGRP‐SA, βGRP3, and βGRP5) and downstream signaling (Dorsal), HSP83 establishes a two‐tiered control that dampens excessive antimicrobial peptide induction during exposure to the native antagonist *Ophiostoma minus* while supporting tolerance toward the mutualist *Leptographium procerum* and limiting excessive Toll pathway activation. These findings provide a mutation‐independent mechanism by which microbial partners can rapidly modulate host immune investment during biological invasions, and they highlight a tractable entry point for manipulating beetle–fungus interactions for biocontrol.

## 5 Experimental Section

### 5.1 Insects, Fungal Strains, and Generation of Surface‐Sterilized Larvae

Adult RTB were collected from *P. tabuliformis* stands in Shanxi Province using pheromone‐baited traps. Beetle pairs were introduced into freshly cut pine bolts to obtain larvae, which were maintained under controlled laboratory conditions [64, 65]. The fungal isolates Lp (CMW 25624) and Om (CMW 26254) were originally obtained from RTB galleries and maintained on malt extract agar [66]. To minimize microbial background, third‐instar larvae were generated using a two‐phase sterilization protocol combining pre‐treatment on sterile artificial diet with daily surface sterilization. Sterility was confirmed by culture‐based assays, and only surface‐sterilized larvae were used for all experiments. All field collections were conducted under permits from the Forest Pests Quarantine and Control Bureau of Shanxi Province, and all animal procedures were approved by the Animal Care and Use Committee of the Institute of Zoology, Chinese Academy of Sciences (Beijing, China). Detailed procedures for beetle husbandry, fungal culture, and larval sterilization are provided in the Supplementary Methods.

### 5.2 Fungal Exposure and Survival Assays

Surface‐sterilized third‐instar larvae were randomly assigned to four treatments (PBS, Lp, Om, and L&O) and exposed by immersion in conidial suspensions. After exposure, larvae were maintained under controlled laboratory conditions and monitored daily for 6 d to assess survival. For RNAi experiments, larvae were injected with dsHSP83 or dsEGFP 48 h before fungal challenge and monitored using the same procedure. Survival data were analyzed using Kaplan–Meier curves with log‐rank tests. The immersion procedure provided a standardized approximation of contact with fungal propagules in colonized galleries and was not used to infer tissue invasion or systemic infection. Detailed conidial preparation, immersion parameters, and larval husbandry procedures are provided in the Supplementary Methods.

### 5.3 RNA Extraction, RNA‐seq, and Differential Expression Analysis

Total RNA was extracted from RTB larvae using TRIzol, with each biological replicate consisting of pooled tissues from three larvae. RNA‐seq libraries were prepared from three biological replicates per treatment and sequenced on an Illumina NovaSeq 6000 platform. Reads were mapped to the RTB genome, and transcript abundance was quantified accordingly [65]. Differentially expressed genes were defined using fold change > 1.5 or < 0.67 for immune profiling analyses and fold change > 2 or < 0.5 for *HSP83* knockdown experiments, with *p* < 0.05 as the significance threshold. KEGG pathway enrichment and clustering analyses were performed using an online bioinformatics platform [67]. Full extraction procedures, sequencing workflows, quality control criteria, and computational parameters are provided in the Supplementary Methods.

### 5.4 qRT‐PCR and Western Blotting

For qRT‐PCR, total RNA was extracted from pooled tissues of three larvae per biological replicate and reverse‐transcribed to cDNA. Gene expression was quantified using a SYBR Green–based detection method, with *rpL39* and *rpL32* serving as internal reference genes for RTB larvae and S2 cells, respectively [68]. Three biological replicates were analyzed for each treatment. For Western blotting, proteins were extracted from pooled tissues of three larvae per replicate, separated by SDS–PAGE, and detected with antibodies against PGRP‐SA, PGRP‐SC2, βGRP3, βGRP5, Dorsal, or HSP83, using β‐tubulin as a loading control. Antibody sources, protein extraction procedures, and complete immunoblot protocols are provided in the Supplementary Methods.

### 5.5 Recombinant Proteins, PRR–Fungus Binding Assays, and GST Pull‐Down

Recombinant DvPGRP‐SA, DvPGRP‐SC2, DvβGRP3, DvβGRP5, DvDorsal, DvRelish2, wild‐type DvHSP83, and three DvHSP83 alanine substitution variants were expressed in *E. coli* and affinity‐purified according to the tag carried by each construct. For PRR–fungus binding assays, purified proteins were incubated with Lp, Om, or L&O conidia, and bound fractions were detected by immunoblotting. To assess PRR–fungus association in the presence of DvHSP83, PRRs were pre‐mixed with recombinant DvHSP83 before exposure to fungal conidia. Direct interactions between HSP83 and immune‐related proteins were examined using GST pull‐down assays, with HSP83–GST as bait and V5‐tagged proteins as prey. Associations of the DvHSP83 variants with selected PRRs and their effects on PRR recovery in fungus‐bound fractions were assessed using the corresponding pull‐down and fungus‐binding assays. Detailed expression conditions, purification procedures, binding assay parameters, and pull‐down workflows are provided in the Supplementary Methods.

### 5.6 RNA Interference and HSP83 Protein Supplementation

For RNA interference, larvae were injected with dsHSP83 or dsEGFP and maintained for 48 h before fungal challenge. Knockdown efficiency was assessed by qRT‐PCR and Western blotting using pooled tissues from three larvae per replicate. For HSP83 supplementation, purified recombinant HSP83 or control protein was injected into larvae, followed by PBS or Om treatment, and samples were collected for gene expression analysis at 96 h after treatment. Injection procedures, dsRNA synthesis methods, protein preparation, and all experimental parameters are described in detail in the Supplementary Methods.

### 5.7 Immunofluorescence and Fluorescence Imaging

For immunofluorescence, paraffin‐embedded sections of RTB larvae were stained with antibodies against HSP83, Dorsal, or the PRRs PGRP‐SA, βGRP3, and βGRP5, followed by fluorescently labeled secondary antibodies. Wheat germ agglutinin (WGA) was used to visualize the plasma membrane, and nuclei were counterstained with DAPI. Stained sections were imaged using a fluorescence scanning platform under identical acquisition settings for all treatments. Complete staining procedures, antigen retrieval conditions, antibody sources, and imaging parameters are provided in the Supplementary Methods.

### 5.8 Co‐IP and DIA Proteomics

For co‐immunoprecipitation, hemolymph and fat body tissues from RTB larvae were homogenized and incubated with an anti‐HSP83 antibody to isolate endogenous HSP83‐associated protein complexes. Co‐IP assays were performed across both RNAi treatments and multiple fungal treatment conditions. Immunoprecipitated samples were subjected to data‐independent acquisition (DIA) mass spectrometry for protein identification and quantification. Pathway enrichment and comparative analyses were conducted to characterize condition‐dependent variation in the composition of HSP83‐associated protein complexes. Detailed Co‐IP procedures, fungal treatment and RNAi sample sets, protein preparation steps, mass spectrometry settings, and DIA data processing parameters are provided in the Supplementary Methods.

### 5.9 Cell Culture and S2 Cell Transfection Assay

*Drosophila* S2 cells were maintained under standard serum‐free conditions and used for heterologous expression assays. Full‐length DvDorsal and DvHSP83 coding sequences were cloned into V5‐tagged expression vectors and transiently transfected into S2 cells, either individually or in combination, to assess Dorsal‐dependent AMP gene induction. Neither Lp nor Om was introduced into the S2 cell cultures. Cells were collected 48 h after transfection for RNA extraction and qRT‐PCR analysis. Detailed culture conditions, plasmid construction procedures, transfection workflows, and primer sequences are provided in the Supplementary Methods.

### 5.10 Phylogenetic and Network Analyses

Phylogenetic analysis of HSP83 was performed using multiple sequence alignment followed by maximum‐likelihood tree construction. Conserved motifs and domain architectures were examined using standard annotation tools. Protein–protein interaction networks were generated from differentially expressed genes using the STRING database and visualized in Cytoscape, and hub genes were identified with CytoHubba. Complete sequence lists, alignment settings, model selection procedures, and network analysis parameters are provided in the Supplementary Methods.

### 5.11 Statistical Analysis

All statistical analyses were performed using Microsoft Excel 2021 and GraphPad Prism 10. Kaplan–Meier survival curves were generated, and statistical significance was evaluated using the log‐rank (Mantel–Cox) test. Hierarchical clustering was conducted with the pheatmap package in R v4.4.0. The Shapiro–Wilk test was used to assess the normality or lognormality of qPCR data across treatments. For comparisons among multiple groups, one‐way ANOVA followed by Dunnett's post hoc test was applied when equal variances were confirmed. Data are presented as mean ± SEM unless stated otherwise. Significance thresholds were denoted as follows: *p < 0.05; **p < 0.01; ***p < 0.001; ****p < 0.0001. Each treatment included three independent biological replicates, and additional statistical details are provided in the figure legends.

## Author Contributions

J.H.S., L.K., and Z.Z. conceived the study. Q.Y., Z.L., L.X., Z.Z., L.K., and J.H.S. developed the methodology. Q.Y. and D.Y. curated the data. Q.Y., Z.L., Y.Y., and J.S. performed the investigation, and Q.Y., Y.Y., and J.S. validated key findings. Q.Y., D.Y., and L.X. conducted formal analyses. Z.Z., L.K., and J.H.S. supervised the project; Z.Z. and J.H.S. acquired funding; and J.H.S. administered the project. Z.L. and J.H.S. provided resources. Q.Y. and D.Y. wrote the original draft, and Q.Y., Z.L., Z.Z., L.K., and J.H.S. reviewed and edited the manuscript. All authors read and approved the final manuscript.

## Funding

This work was supported by the National Natural Science Foundation of China (32088102, 32061123002), the Initiative Scientific Research Program of the Institute of Zoology, Chinese Academy of Sciences (2024IOZ0105), the Hebei Natural Science Foundation (C2022201042), the 2026 Open Research Fund of NHC Key Laboratory of Tropical Disease Control, Hainan Medical University (No. 2026NHCTDC11001), and the High‐level Talent Research Funding Project of Hebei University (2023HBQZYCXY003).

## Conflicts of Interest

The authors declare no conflicts of interest.

## Supporting information




**Supporting File**: advs77421‐sup‐0001‐SuppMat.docx.

## Data Availability

RNA‐seq data have been deposited in the NCBI Sequence Read Archive under accession PRJNA1178106. Proteomics data have been deposited in the ProteomeXchange Consortium via the PRIDE repository under accession PXD069324. All other data supporting the findings of this study are available within the article and its Supplementary Information files or from the corresponding author upon reasonable request.
