# Supplementary material for: Symbiont‐Induced HSP83 Establishes Two‐Tiered Control of Antifungal Immunity in an Invasive Beetle–Fungus Complex
Source: Adv Sci (Weinh). 2026 Aug 27;13(63):e77421. doi: 10.1002/advs.77421 (PMC13521185; doi:10.1002/advs.77421)
Supplement: Supplementary file 1 [file ADVS-13-e77421-s001.docx]

Supporting Information

Symbiont-Induced HSP83 Establishes Two-Tiered Control of Antifungal Immunity in an Invasive Beetle–Fungus Complex

Qingtai Yang, Zhudong Liu, Yuqiao Yin, Jing Su, Dunyang Yu, Longsheng Xing, Zhen Zou,* Le Kang,* Jianghua Sun*

1. Supplementary Methods

1.1. Detailed beetle collection, sexing, and colony maintenance

Adult red turpentine beetles (*Dendroctonus valens*) were collected annually between May and June from 2018 to 2025 at Beishe Mountain, Shanxi Province, China (37°51′N, 111°31′E) using pheromone-baited traps. Adult beetles were sexed based on the males’ stridulation behavior and maintained in 10-cm Petri dishes under laboratory conditions. Beetle pairs were introduced into freshly cut Chinese pine (*Pinus tabuliformis*) bolts (30-cm long × 20-cm diameter) to establish galleries and obtain larvae.^[1–2]^ Third-instar larvae, approximately 10 mm in length and collected 45 days after beetle introduction, were used for all experiments.

1.2. Fungal strain isolation, maintenance, and long-term storage

*Leptographium procerum* (CMW 25624, Lp) and *Ophiostoma minus* (CMW 26254, Om) were isolated from the exoskeletons of adult *Dendroctonus valens* and from their galleries in infested *P.* *tabuliformis* trees in Shanxi Province, China. Both fungal species were maintained in the laboratory on malt extract agar (MEA; 2% malt extract, 2% agar) at 25 °C in the dark. Subculturing was performed every four weeks to maintain active cultures. For long-term preservation, fungal stocks were stored in 25% glycerol at −80 °C.

1.3. Generation of surface-sterilized larvae (two-phase protocol)

Larvae were generated using a two-phase sterilization protocol designed to eliminate surface-associated microbes while maintaining larval viability. In the first phase, larvae were removed from pine galleries 14 days before experiments and maintained on sterile artificial diet in an environmental chamber at 25 °C, 55% relative humidity, under a 14:10 h light:dark photoperiod.^[3–4]^ In the second phase, beginning seven days prior to experiments, larvae were subjected to daily surface sterilization using a modified procedure.^[3]^ Larvae were first rinsed with sterile distilled water for 30 s, immersed in 10% commercial bleach (0.6% sodium hypochlorite) for 60 s, and washed three times with sterile distilled water for 30 s each. They were then immersed in 75% ethanol for 60 s and washed three additional times with sterile distilled water. After sterilization, larvae were transferred to sterile artificial diet supplemented with an antibiotic cocktail containing erythromycin (20 µg mL⁻¹), gentamicin (10 µg mL⁻¹), and kanamycin sulfate (50 µg mL⁻¹), and provided with fresh diet daily. Sterility was confirmed by plating larval surface swabs on MEA and LB agar plates and incubating them at 25 °C for 30 days. Dead larvae were removed daily and excluded from subsequent experiments.

1.4. Preparation of conidial suspensions

Lp and Om were cultured on malt extract agar for 28 days to ensure mature conidiation. Plates were flooded with 5 mL of sterile phosphate-buffered saline (PBS; 137 mM NaCl, 2.7 mM KCl, 10 mM Na₂HPO₄, 1.8 mM KH₂PO₄, pH 7.4) containing 0.05% Tween-20, and the surface was gently scraped with a sterile spreader to release conidia.^[5]^ The suspension was filtered through sterile Miracloth (Millipore) to remove hyphal fragments, and conidial concentrations were quantified using a hemocytometer with three independent counts per sample. Working suspensions were adjusted to 1 × 10⁹ conidia mL⁻¹ in PBS.

For mixed inocula (L&O), equal volumes of Lp and Om suspensions at 2 × 10⁹ conidia mL⁻¹ were combined immediately before use to yield a final concentration of 1 × 10⁹ conidia mL⁻¹ for each species. Conidial viability was verified by plating serial dilutions on malt extract agar and assessing germination after 24 h, and only suspensions with viability above 95% were used for fungal-exposure experiments.

1.5. Survival assay detailed procedure

Surface-sterilized third-instar larvae were randomly assigned to four treatment groups (PBS, Lp, Om, and L&O). Larvae were immersed for 40 s in 30 mL of the corresponding treatment suspension. After immersion, excess liquid was aspirated, and the larvae were transferred to sterile artificial diet. Larvae were then monitored daily for six days. Mortality was determined by the absence of movement in response to gentle prodding with a sterile probe. Dead larvae were removed immediately, surface-sterilized, and discarded under sterile conditions.

For RNAi-based survival assays, larvae were pretreated with dsHSP83 or dsEGFP and allowed to recover for 48 h before fungal challenge. Treated larvae were then immersed using the same procedures described above and monitored daily for six days. Each survival assay included at least 38 larvae per group and was independently replicated three times using distinct larval cohorts.

1.6. dsRNA design, synthesis, and injection procedures

A 520 bp fragment from the coding sequence of *DvHSP83* was selected to design primers containing a T7 promoter adapter.^[6]^ Double-stranded RNA was synthesized using the T7 RiboMAX Express RNAi System (Promega) following the manufacturer’s instructions. A dsRNA targeting enhanced green fluorescent protein (GFP) was synthesized in parallel and used as a negative control.

For larval RNA interference, 1 µg of purified dsRNA was injected into the hemocoel of each surface-sterilized third-instar larva using a Nanoliter 2000 injector (World Precision Instruments, Sarasota, FL, USA).^[7]^ After injection, larvae were maintained under standard conditions and used after a 48-h recovery period. All primers used for dsRNA synthesis are listed in Table S1.

1.7. Recombinant HSP83 supplementation experiment

Surface-sterilized third-instar larvae were divided into four treatment groups for recombinant protein supplementation. Two groups were injected with 1 µg of purified recombinant DvHSP83 (HSP83), and the other two groups were injected with 1 µg of EGFP as a control (GFP), using a Nanoliter 2000 injector (World Precision Instruments, Sarasota, FL, USA). After a 48-h recovery period, each injection set was challenged with either PBS or Om conidia, generating the GFP + PBS, GFP + Om, HSP83 + PBS, and HSP83 + Om treatments. Larvae were collected 96 h after treatment for total RNA extraction and subsequent gene-expression analysis.

1.8. RNA extraction, library preparation, sequencing

Tissue samples were homogenized in 1 mL of TRIzol reagent (Invitrogen) for 180 s at 65 Hz using a tissue homogenizer. Homogenates were incubated for 5 min at room temperature and mixed with 200 µL chloroform:isoamyl alcohol (24:1, v/v). Samples were vortexed vigorously for 15 s, incubated for 3 min, and centrifuged at 12,000 × g for 15 min at 4 °C to separate the phases. The RNA-containing aqueous phase was transferred to a new tube and purified using the GeneJET RNA Purification Kit (Thermo Scientific) according to the manufacturer’s protocol. RNA concentration and purity were measured with a NanoDrop 2000 spectrophotometer (Thermo Scientific), ensuring A260/A280 > 1.8 and A260/A230 > 2.0.

RNA-seq libraries were constructed and sequenced by Novogene (Beijing, China) on an Illumina NovaSeq 6000 platform. Reads were aligned to the *D. valens* reference genome, and gene families were annotated using the available reference annotations.^[2]^ RNA-seq libraries were prepared for PBS, Lp, L&O, and Om treatments, with three biological replicates per treatment and three larvae pooled per replicate. For *HSP83*-knockdown experiments, RNA libraries were prepared from iGFP + PBS, iHSP83 + PBS, iHSP83 + Lp, iHSP83 + L&O, and iHSP83 + Om treatments, each with three biological replicates. Differentially expressed genes were defined as transcripts with fold change > 1.5 or < 0.67 and *P* < 0.05 for fungal immune profiling, or fold change > 2 or < 0.5 and *P* < 0.05 for *HSP83*-knockdown analyses.

1.9. qRT-PCR

Total RNA (1 µg per sample) was reverse-transcribed into cDNA using the PrimeScript RT Reagent Kit with gDNA Eraser (Takara) according to the manufacturer’s protocol. Quantitative PCR was performed using TB Green Premix Ex Taq II (Takara) in a 20 µL reaction mixture containing 10 µL TB Green Premix Ex Taq II, 0.8 µL of each primer, 2 µL diluted cDNA template, and 6.4 µL nuclease-free water. qPCR assays were conducted on an Applied Biosystems QuantStudio system (Thermo Fisher Scientific) under the following cycling program: 95 °C for 30 s, followed by 40 cycles of 95 °C for 5 s and 60 °C for 30 s. Three biological replicates were analyzed for each treatment. Primer sequences are listed in Table S1.

1.10. Total protein extraction and Western blotting

Larval tissues from three individuals per treatment were homogenized on ice in RIPA strong lysis buffer (Cwbio) supplemented with a 10× protease inhibitor cocktail (Thermo Fisher Scientific) at a ratio of 0.001 g tissue per 10 µL buffer and incubated for 30 min.^[8]^ Lysates were centrifuged at 12,000 rpm for 15 min at 4 °C, and protein concentrations were determined using a BCA Protein Assay Kit (Cwbio).^[9]^ Equal amounts of total protein were loaded for electrophoresis.

Proteins were separated on 4–20% gradient Tris–Glycine gels (EASYBIO) at 180 V for 45 min and transferred onto polyvinylidene difluoride membranes (Millipore) at 400 mA for 40 min. Membranes were blocked with StartingBlock T20 (TBS) blocking buffer (Thermo Scientific) for 1 h at room temperature, incubated with primary antibodies overnight at 4 °C, and then incubated with HRP-conjugated secondary antibodies for 1 h at room temperature. Signal detection was performed using SuperSignal West Plus chemiluminescent substrate (Thermo Scientific).

Primary antibodies included rabbit polyclonal anti-PGRP-SA, anti-PGRP-SC2, anti-βGRP3, anti-βGRP5, anti-Dorsal, and anti-HSP83 (all generated in this study), mouse monoclonal anti-Hsp90 (Abcam, ab59459), rabbit monoclonal anti-β-Tubulin (Absin, abs171597), mouse monoclonal anti-GST (EASYBIO, BE2065), and mouse monoclonal anti-V5 (Invitrogen, R960-25). Secondary antibodies included goat anti-rabbit IgG and goat anti-mouse IgG (EASYBIO).

1.11. Complex modeling with AlphaFold Server and selection of candidate DvHSP83 residues for mutagenesis

Complex models were generated using AlphaFold Server (Google DeepMind; powered by AlphaFold 3; accessed April 2026).^[10]^ To represent the canonical dimeric architecture of proteins in the HSP90 family, each modeling input comprised two full-length DvHSP83 molecules and one pattern recognition receptor (PRR). Separate predictions were performed for DvPGRP-SA, DvβGRP3, DvβGRP5, and DvPGRP-SC2. In each predicted complex, chains A and B represented the two DvHSP83 molecules, whereas chain C represented the corresponding PRR. The top-ranked model returned by the server was used for subsequent analysis. The predicted TM score (pTM) and interface predicted TM score (ipTM) were recorded as measures of overall model confidence and interchain-interface confidence, respectively. Because the ipTM values indicated limited confidence in the precise relative arrangement of the modeled partners, the models were used only for exploratory comparison across models and prioritization of candidate residues.

Predicted complexes were inspected using The PyMOL Molecular Graphics System (Schrödinger, LLC). Candidate contacts were identified using static criteria based on interatomic distance. Candidate residues involved in polar contacts were defined as DvHSP83 residues containing nitrogen or oxygen atoms located within 3.5 Å of nitrogen or oxygen atoms in the PRR. Candidate residues involved in hydrophobic contacts were defined as hydrophobic DvHSP83 residues containing carbon atoms located within 5.0 Å of carbon atoms in hydrophobic PRR residues. The set of hydrophobic residues comprised Ala, Val, Leu, Ile, Met, Pro, Phe, Trp, and Tyr. Candidate residues were recorded separately for DvHSP83 chains A and B.

DvPGRP-SC2, which showed no detectable association with wild-type DvHSP83 in GST pull-down assays, was included as a nonbinding specificity comparator. Candidate DvHSP83 residues were manually compared across the four top-ranked complex models. Residues also recovered in the DvHSP83–DvPGRP-SC2 model were not prioritized as candidates specific to the interacting models. E15 was recurrently identified in the predicted complexes containing DvPGRP-SA, DvβGRP3, and DvβGRP5, whereas E61 and K64 were shared between the DvβGRP3 and DvβGRP5 models. These residues were therefore selected for alanine substitution analysis. Model confidence scores and the prioritization across models of candidate DvHSP83 residues are summarized in Table S2.

The predicted structures were used solely to prioritize candidate residues for subsequent experimental analysis and were not interpreted as experimentally resolved atomic interfaces.

1.12. Recombinant protein expression and purification

The full-length coding sequences of *DvPGRP-SA*, *DvPGRP-SC2*, *DvβGRP3*, *DvβGRP5*, *DvDorsal*, *DvRelish2*, and *DvHSP83* were cloned into pET-28a vectors containing His and V5 tags through de novo gene synthesis. The full-length coding sequence of *DvHSP83* was also inserted into a pGEX-4T-1 vector containing a GST tag. Three alanine substitution variants of DvHSP83 were generated based on the residue prioritization using AlphaFold Server: DvHSP83-E15A (HSP83-1Mut), DvHSP83-E61A/K64A (HSP83-2Mut), and DvHSP83-E15A/E61A/K64A (HSP83-3Mut). The corresponding coding sequences were commercially synthesized and inserted separately into pET-28a vectors carrying His and V5 tags and into pGEX-4T-1 vectors carrying a GST tag. All constructs were verified by sequencing.

Constructs were amplified in *Escherichia coli* DH5α and transformed into *E. coli* BL21(DE3) for protein expression. Recombinant proteins were expressed and purified following previously described procedures.^[11]^ After induction, cells were lysed, and supernatants were subjected to affinity purification using Ni–NTA agarose (Qiagen) or Glutathione Sepharose 4B (GE Healthcare), depending on the fusion tag. Protease Inhibitor Cocktail (Beijing Solarbio Science & Technology Co., Ltd.) was added to all lysis and purification buffers throughout the procedure to minimize proteolytic degradation. Protein concentrations were determined using a BCA Protein Assay Kit (Cwbio), and purified proteins were aliquoted and stored at −80 °C until use.

1.13. PRR–fungus binding assays

Before incubation, 1 × 10⁶ conidia of Lp, Om, or L&O were pelleted at 3,000 × *g* for 3 min, washed twice with buffer D (20 mM Tris-HCl, 20 mM NaCl, pH 7.5), and once with TBS buffer (25 mM Tris-HCl, 137 mM NaCl, 2.7 mM KCl, pH 7.6). Recombinant DvPGRP-SA, DvPGRP-SC2, DvβGRP3, or DvβGRP5 (2.5 µg) was then incubated with the conidia in 50 µL buffer D, with PBS in place of conidia serving as a negative control.^[12–13]^ To assess PRR recovery in fungus-bound fractions in the presence of DvHSP83, DvPGRP-SA, DvβGRP3, or DvβGRP5 was pre-mixed with an equal amount of DvHSP83 (2.5 µg each) before incubation with the conidia.

After incubation for 1 h at 4 °C, conidia were pelleted by centrifugation at 10,000 × *g* for 15 min at 4 °C, washed three times with 200 µL TBS, and resuspended in 40 µL buffer D. Protein–fungus complexes were analyzed by SDS–PAGE and immunoblotting using a mouse monoclonal anti-V5 antibody (Invitrogen, R960-25).

1.14. Fungus-binding assays using DvHSP83 variants

Two sets of fungus-binding assays were performed using His/V5-tagged DvHSP83 variants. In the first set, 2.5 µg of HSP83-1Mut, HSP83-2Mut, or HSP83-3Mut was incubated separately with 1 × 10⁶ conidia of Lp, Om, or L&O. PBS without fungal conidia was included as a negative control.

In the second set, 2.5 µg of His/V5-tagged DvPGRP-SA, DvβGRP3, or DvβGRP5 was pre-mixed with an equal mass of the indicated DvHSP83 variant before exposure to 1 × 10⁶ conidia of Lp, Om, or L&O. PBS without fungal conidia was included as a negative control.

Incubation, washing, centrifugation, and recovery of fungus-bound fractions were performed as described above for the binding assays with the wild-type protein. Input and fungus-bound fractions were resolved by SDS–PAGE and detected by immunoblotting using an anti-V5 monoclonal antibody. Signals from DvHSP83 variants and PRRs were distinguished according to their respective molecular masses. All assays were independently repeated three times.

1.15. GST pull-down assays

GST pull-down assays were performed using purified recombinant proteins to assess direct interactions between DvHSP83 and selected immune proteins.^[14]^ The DvHSP83–GST fusion protein served as the bait, and GST alone expressed from the empty pGEX-4T-1 vector was used as a negative control. The six purified V5-tagged prey proteins were DvPGRP-SA, DvPGRP-SC2, DvβGRP3, DvβGRP5, DvDorsal, and DvRelish2. They were expressed from pET-28a vectors, with the V5-tag protein expressed from the empty pET-28a vector used as an additional negative control. In addition, GST pull-down assays were performed to assess the association of the DvHSP83 variants with selected PRRs. GST-tagged HSP83-1Mut, HSP83-2Mut, and HSP83-3Mut were used as bait proteins. GST purified from the empty pGEX-4T-1 vector was used as a bait control. His/V5-tagged DvPGRP-SA, DvβGRP3, DvβGRP5, and DvPGRP-SC2 were used as prey proteins, and the product purified from the empty pET-28a vector was included as an additional prey control.

For each reaction, 50 µL Glutathione Sepharose 4B agarose (GE Healthcare) was equilibrated in PBS and preloaded with 250 µg of the corresponding purified GST-tagged bait protein by incubation for 30 min at 4 °C. An amount of each V5-tagged prey protein corresponding to a twofold molar excess relative to the bait was then added and incubated for 1 h at 4 °C with gentle agitation. Beads were washed eight times with PBS to remove nonspecific interactions and eluted for downstream Western blot analysis. Anti-GST and anti-V5 monoclonal antibodies (EASYBIO and Invitrogen, respectively) were used to verify input and co-precipitated proteins. The assays were independently repeated three times.

1.16. Immunofluorescence staining and fluorescence imaging

Larvae were dehydrated through a 70–100% ethanol gradient, embedded in paraffin, and sectioned at 4 µm thickness using a Leica RM2235 microtome. Sections were dried at 65 °C for 1 h and subjected to antigen retrieval by microwave heating in retrieval buffer for 15 min, followed by cooling to room temperature. Slides were blocked with 3% bovine serum albumin (BSA) for 30 min at room temperature and incubated overnight at 4 °C with primary antibodies (1:200), including rabbit polyclonal anti-PGRP-SA, anti-PGRP-SC2, anti-βGRP3, anti-βGRP5, anti-Dorsal, and anti-HSP83 (all generated in this study), and mouse monoclonal anti-Hsp90 (Abcam, ab59459). After three washes with PBS, sections were incubated with fluorescently labeled secondary antibodies (1:300), including Goat Anti-Mouse IgG (H+L) Alexa Fluor 594 (Abcam, ab150116), Donkey Anti-Rabbit IgG (H+L) Alexa Fluor 647 (Abcam, ab150075), and Goat Anti-Rabbit IgG (H+L) Alexa Fluor 594 (Abcam, ab150080), for 30 min at 37 °C in the dark.

To visualize the plasma membrane, sections were stained with Wheat Germ Agglutinin (WGA; Thermo Fisher Scientific, Alexa Fluor 488 conjugate, W11261, 1:300) for 30 min, followed by DAPI staining (Macklin, 1:10,000) for 10 min. Slides were mounted in antifade aqueous mounting medium and imaged using a KFBIO KF-PRO-020 fluorescence scanner under identical exposure settings.

For Dorsal–HSP83 co-localization, larvae exposed to Om or L&O were stained using rabbit anti-Dorsal polyclonal antibody and mouse anti-HSP83 monoclonal antibody. For HSP83 localization, PBS- or L&O-exposed larvae were stained using rabbit anti-HSP83 polyclonal antibody with WGA labeling the plasma membrane. For PRR–HSP83 co-localization, larvae exposed to Om or L&O were stained with rabbit anti-βGRP3, anti-βGRP5, or anti-PGRP-SA polyclonal antibodies together with mouse anti-HSP83 monoclonal antibody.

1.17. Co-IP and DIA proteomics

Co-immunoprecipitation (Co-IP) assays were conducted following established protocols.^[15]^ Mixed hemolymph and fat body tissues were collected from surface-sterilized third-instar larvae subjected to RNAi and fungal treatment conditions, including iGFP + PBS, iGFP + L&O, iHSP83 + PBS, and iHSP83 + L&O. Each treatment contained three biological replicates, with five larvae pooled per replicate. Tissues were lysed on ice for 30 min in RIPA Lysis Buffer (Weak) (Beyotime) supplemented with a 10× protease inhibitor cocktail (Thermo Fisher Scientific). Lysates were precleared with 50 µL Protein A–Sepharose (Roche) for 1 h at 4 °C to remove nonspecific binding. The supernatant was then incubated with 10 µL rabbit anti-HSP83 polyclonal antibody for 1 h at 4 °C, followed by addition of 50 µL Protein A–Sepharose and overnight incubation at 4 °C with gentle agitation.

Protein A–bound immune complexes were collected by centrifugation, washed six times with washing buffer, and resuspended in 60 µL protein loading buffer for downstream mass spectrometry analysis. A parallel set of Co-IP assays using anti-HSP83 polyclonal antibody was performed on PBS-, Lp-, L&O-, and Om-exposed larvae to assess treatment-dependent interactions.

Mass spectrometry–based proteomic analysis of co-immunoprecipitated samples was performed using a data-independent acquisition (DIA) strategy.^[16]^ Samples were embedded in polyacrylamide gels for protein extraction, purified, and digested with trypsin. The resulting peptides were separated on a Vanquish Neo liquid chromatography system (Thermo Fisher Scientific) coupled to an Astral mass spectrometer (Thermo Fisher Scientific) operating in DIA mode. A spectral library was generated from DIA data combined with the *D. valens* peptide sequence database using DIA-NN (version 1.8.1).^[17]^ Protein quantification was extracted using Spectronaut (Biognosys). Peptide concentrations and total peptide amounts for each DIA sample are listed in Table S3.

Proteins showing differential abundance in the HSP83 Co-IP fractions were identified using a fold change threshold of > 1.4 or < 0.71 with *P* < 0.05. Bioinformatic analyses and visualization were conducted using the online platform (<http://www.bioinformatics.com.cn>). Raw proteomics files have been deposited in the ProteomeXchange Consortium via the PRIDE partner repository under accession PXD069324.^[18]^

1.18. S2 cell culture, transfection, and RNA/protein isolation

*Drosophila* S2 cells were maintained in Serum-Free Insect Cell Culture Medium (Cytiva) without antibiotics at 27 °C in the absence of CO₂ and were subcultured every 3–4 days at a split ratio of 1:5.^[19–20]^ Transfections were performed using full-length *DvHSP83* and *DvDorsal* cDNAs cloned into V5-tagged pAc5.1b expression vectors. For each transfection, 1 µg plasmid DNA was mixed with Cellfectin II Reagent (Gibco) at a 1:3 DNA:reagent ratio following the manufacturer’s protocol. For co-transfections, 0.5 µg of each plasmid was used. A pAc5.1b-GFP plasmid was used to monitor transfection efficiency, and only experiments with >80% efficiency were processed further.

Cells were harvested 48 h after transfection for RNA or protein extraction using RIPA Lysis Buffer (Strong) (Cwbio). Cell viability (>90%) was verified by trypan blue exclusion. All transfection experiments were performed in triplicate using independent S2 cell passages.

1.19. Phylogenetic analysis and conserved domain annotation

To identify the candidate *D. valens* HSP83 (evm.model.scaffold_10.111) and determine its evolutionary position within the heat shock protein superfamily, a total of 122 protein sequences were compiled. The dataset comprised five components: the candidate *D. valens* HSP83; 86 *Drosophila melanogaster* HSP sequences retrieved from FlyBase (FlyBase ID: FBgg0000501), retaining only the longest isoform for each gene;^[21]^ 11 representative insect HSP83 orthologs, including three Coleopteran (*Tribolium castaneum*, *Anoplophora glabripennis*, *Agrilus planipennis*; NCBI accessions NP_001300806.1, XP_018579855.1, XP_018325773.1), three Lepidopteran (*Bombyx mori*, *Spodoptera frugiperda*, *Plutella xylostella*), two Dipteran (*Anopheles gambiae*, *Aedes aegypti*), and three Hymenopteran species (*Apis mellifera*, *Vespa mandarinia*, *Solenopsis invicta*); eight mammalian HSP90 family members from *Homo sapiens* (HSP90α, HSP90β, GRP94, TRAP1) and *Mus musculus* (Hsp90α, Hsp90β, Grp94, Trap1); and 16 plant orthologs from *Arabidopsis thaliana* and *Oryza sativa*.^[22–24]^

Multiple sequence alignment was conducted using MAFFT v7.526 with parameters “--localpair --maxiterate 1000”.^[25]^ The optimal amino acid substitution model was determined using ModelFinder in IQ-TREE v2.3.6 based on the Bayesian Information Criterion (BIC).^[26]^ Phylogenetic trees were inferred by maximum-likelihood analysis in IQ-TREE v2.3.6 with parameters “-B 5000 -bnni” and visualized using iTOL (<https://itol.embl.de/>).^[27]^

To assess sequence conservation, 13 representative HSP83 proteins (including *D. valens* HSP83 and orthologs from mammals and plants) were analyzed using the NCBI Conserved Domain Database (CDD) with a default E-value threshold of 0.01.^[28–29]^ Theoretical molecular weights were computed using ProtParam.^[30]^ Multiple sequence alignment was additionally used to verify the presence of characteristic conserved motifs, including the C-terminal pentapeptide MEEVD and lysine residues K270 and K277, and results were visualized using the ESPript 3.0 server.^[31]^

1.20. Construction of a PPI network and identification of hub genes

Immunity-related differentially expressed genes showing concordant regulation in Lp- and L&O-exposed larvae relative to PBS controls (log₂FC > 0 in both comparisons or log₂FC < 0 in both comparisons) were uploaded to the STRING database (<https://string-db.org/>) to obtain predicted protein–protein interactions. Interaction data were exported and visualized using Cytoscape v3.10.2. Hub genes were identified using the CytoHubba plugin in Cytoscape by applying 11 topological algorithms, including ClusteringCoefficient, Degree, EdgeBetweenness, BetweennessCentrality, AverageShortestPathLength, NeighborhoodConnectivity, TopologicalCoefficient, ClosenessCentrality, Eccentricity, Stress, and Radiality.^[32]^ The expression profiles of the seven resulting hub genes were subsequently examined by hierarchical clustering across PBS, Lp, L&O, and Om treatments.

2. Supplementary Figures S1–S14

**Figure S1.** Toll pathway activation in RTB larvae following Om treatment, related to Figure 1**.** A) Expression patterns of 93 genes from the Toll and IMD signaling pathway among PBS, Lp, L&O and Om treated RTB transcriptome data at 96 h after conidial exposure are shown in a heatmap. Values were the mean expression of each gene among three replicates centered and scaled in the row direction with −1.5 to 1.5 corresponding to the blue and red colors. Genes discussed in the text are highlighted in red. B) qRT-PCR measured the transcription levels of four selected pattern recognition receptors in RTB at 96 h after conidial exposure with Lp (green bars), L&O (purple bars) and Om (red bars) relative to PBS (control, blue bars, represented as 1). The y axis shows relative fold changes normalized to internal control *rpL39*. Selected pattern recognition receptors were not significantly regulated after Lp, L&O, or Om treatment. One-way ANOVA followed by Dunnett's multiple comparisons test was used for statistical analysis. Error bars represent means ± SEM of three independent experiments. ns, not significant. C) qRT-PCR detected the expression of three selected Serpins (Toll signaling pathway inhibitors) in RTB at 96 h after conidial exposure with Lp (green bars), L&O (purple bars) and Om (red bars) against PBS (control, blue bars, represented as 1). The y axis shows relative fold changes normalized to internal control *rpL39*. Selected Serpins were not significantly regulated after Lp, L&O or Om treatment. One-way ANOVA followed by Dunnett's multiple comparisons test was used for statistical analysis. Error bars represent means ± SEM of three independent experiments. ns, not significant.

**Figure S2.** Phylogenetic and structural analysis of DvHSP83, related to Figure 2. A) Phylogenetic tree of 122 representative heat shock protein (HSP) sequences from mammals, plants, and insects. The tree was constructed using maximum likelihood methods and visualized using iTOL. DvHSP83 is highlighted in red. B) Structural analysis of DvHSP83 showed its characteristic HSP90 architecture. DvHSP83 is highlighted in red. C) Theoretical molecular weight of DvHSP83. DvHSP83 is highlighted in red.

**Figure S3.** *HSP83* was efficiently knocked down in RTB, and the Toll pathway was activated after depletion of *HSP83*, related to Figure 3. A) Heatmap of 22 heat shock proteins in RTB transcriptomes after dsHSP83 injection (iHSP83) followed by exposure to PBS (iHSP83+PBS), Lp (iHSP83+Lp), L&O (iHSP83+L&O) or Om (iHSP83+Om), and after dsEGFP injection with PBS (iGFP+PBS). Values are mean expression from three replicates, centered and scaled per row (blue, −1.5; red, 1.5). HSP83 is indicated in red; no other HSPs were affected by *HSP83* interference. B) Circle diagram summarizing 51 Toll and IMD pathway members in iHSP83+Lp, iHSP83+L&O, iHSP83+Om and iGFP+PBS, all compared with iHSP83+PBS. Upregulated genes are marked in red, downregulated in blue. Inner-ring height reflects −log₁₀(*P* value); orange trapezoids indicate significantly upregulated pathways, white indicates non‑significant regulation. C) Heatmap showing 93 Toll and IMD pathway genes in the same treatments as in panel A. Data are displayed as in panel A; genes discussed in the text are highlighted in red. D) Volcano plot of 41 IMD‑pathway genes in iHSP83+Om versus iHSP83+PBS. The x axis shows log₂(fold change) with thresholds FC > 2 (right vertical dashed line) and FC < 0.5 (left vertical dashed line); the y axis shows −log₁₀(*P* value) with a threshold of *P* < 0.05 (horizontal dashed line). Transcripts that are significantly up- or downregulated (FC > 2 or < 0.5, *P* < 0.05) are shown in red; transcripts with significant but smaller changes (0.5 ≤ FC ≤ 2, *P* < 0.05) are shown in blue; transcripts with large but non‑significant changes (FC > 2 or < 0.5, *P* > 0.05) are shown in green; all remaining transcripts are shown in gray. Three significantly upregulated genes are highlighted. iHSP83 + PBS served as the reference condition. Statistical significance was assessed using a two‑sided unpaired t‑test.

**Figure S4.** Histological validation and mutagenesis guided by AlphaFold Server identifies E15 as a functionally important determinant of the association between DvHSP83 and PRRs, related to Figure 4. A) Hematoxylin and eosin staining images from cross-sections of treated RTB larvae in Figure 4A show hemocytes and fat cells in RTB larvae. Nuclei are stained blue with hematoxylin, and the cytoplasm is stained pink with eosin. Hemocytes discussed in the text are highlighted by filled blue triangles. Fat cells discussed in the text are highlighted by filled dark triangles. Scale bar: 10 μm. B) GST pull-down assays assessing the association of GST-tagged DvHSP83 variants with His/V5-tagged PRRs. Both E15A-containing variants (HSP83-1Mut and HSP83-3Mut) showed no detectable co-precipitation with DvPGRP-SA, DvβGRP3, or DvβGRP5. In contrast, HSP83-2Mut retained detectable association with all three PRRs. None of the DvHSP83 variants showed detectable association with DvPGRP-SC2 or the product purified from the empty pET-28a vector. GST purified from the empty pGEX-4T-1 vector served as the bait control. Input and output fractions were detected using anti-GST and anti-V5 antibodies. C) Direct binding of His/V5-tagged DvHSP83 variants to fungal conidia. HSP83-1Mut, HSP83-2Mut, and HSP83-3Mut retained detectable binding to L&O and Om conidia, whereas no detectable binding was observed with Lp conidia or the PBS control. Input and fungus-bound fractions were detected using an anti-V5 antibody. D) PRR–fungus binding assays following pre-mixing with the indicated DvHSP83 variants. Following pre-mixing with the variants containing E15A, DvPGRP-SA, DvβGRP3, and DvβGRP5 remained readily detectable in L&O- and Om-bound fractions. In contrast, pre-mixing with DvHSP83-E61A/K64A qualitatively reduced PRR recovery in the corresponding fungus-bound fractions. Signals from DvHSP83 variants and PRRs were distinguished according to their respective molecular masses. Input and fungus-bound fractions were detected using an anti-V5 antibody. E) Hematoxylin and eosin staining images from cross-sections of treated RTB larvae in Figure 4E show hemocytes and fat cells in RTB larvae. Nuclei are stained blue with hematoxylin, and the cytoplasm is stained pink with eosin. Hemocytes discussed in the text are highlighted by filled blue triangles. Fat cells discussed in the text are highlighted by filled dark triangles. Scale bar: 10 μm. Blots are representative of three independent experiments. Original, unaltered full blot images corresponding to panels B, C, and D are provided in Figure S10, S11, and S12–S14, respectively.

**Figure S5.** Differential protein abundance in anti-HSP83 Co-IP fractions and histological analysis of RTB larvae following fungal treatments, related to Figure 5. A) Volcano plots of 953 proteins detected by DIA proteomics in anti-HSP83 Co-IP fractions from RTB larvae following Lp, L&O, Om, or PBS treatment. Each dot represents one protein. The x axis shows log₂(fold change) with thresholds FC > 1.4 (right vertical dashed line) and FC < 0.71 (left vertical dashed line); the y axis shows −log₁₀(*P* value) with a threshold of *P* < 0.05 (horizontal dashed line). Proteins showing significantly higher or lower abundance in the anti-HSP83 Co-IP fractions (FC > 1.4 or < 0.71, *P* < 0.05) are in red; proteins with large but non-significant abundance differences (FC > 1.4 or < 0.71, *P* > 0.05) are in green; all remaining proteins are shown in gray. Candidate proteins HSP83 and Dorsal discussed in the text are highlighted (filled black triangles). In each comparison, the second group served as the reference condition. Statistical significance was determined using a two‑sided unpaired t‑test. The four comparisons were Lp vs PBS, L&O vs PBS, Om vs PBS, and Om vs L&O. B) Hematoxylin–eosin staining of cross‑sections from treated RTB larvae corresponding to Figure 5D shows hemocytes and fat cells. Nuclei are stained blue with hematoxylin, and the cytoplasm is stained pink with eosin. Hemocytes discussed in the text are indicated by filled blue triangles and fat cells by filled dark triangles. Scale bar, 10 μm.

**Figure S6.** Original, unaltered full blot images corresponding to Figure 1E and 1F. A) Original full blot images corresponding to Figure 1E, showing Dorsal, PGRP-SA, PGRP-SC2, βGRP3, βGRP5, and β-tubulin in RTB larvae at 96 h after exposure to PBS, Lp, L&O, or Om. B) Original full blot images corresponding to Figure 1F, showing the input and fungus-bound output fractions for DvPGRP-SA, DvPGRP-SC2, DvβGRP3, and DvβGRP5 in binding assays with PBS, Lp, L&O, or Om.

**Figure S7.** Original, unaltered full blot images corresponding to Figure 2E, Figure 2G, and Figure 3F. A) Original full blot images corresponding to Figure 2E, showing HSP83 and β-tubulin in RTB larvae at 96 h after exposure to PBS, Lp, L&O, or Om. B) Original full blot images corresponding to Figure 2G, showing HSP83 and β-tubulin after dsEGFP or dsHSP83 treatment under PBS or Om exposure. C) Original full blot images corresponding to Figure 3F, showing Dorsal, PGRP-SA, PGRP-SC2, βGRP3, βGRP5, and β-tubulin in dsEGFP- or dsHSP83-treated RTB larvae under PBS or Om exposure.

**Figure S8.** Original, unaltered full blot images corresponding to Figure 4B. Input and pull-down output fractions from GST pull-down assays examining the association of DvHSP83 with selected immune proteins. Bait and prey proteins were detected using anti-GST and anti-V5 antibodies, respectively.

**Figure S9.** Original, unaltered full blot images corresponding to Figure 4C and 4D. A) Input and fungus-bound output fractions of DvHSP83 in fungus-binding assays with PBS, Lp, L&O, or Om. B) Input and fungus-bound output fractions from PRR–fungus binding assays following pre-mixing of DvHSP83 with DvPGRP-SA, DvβGRP3, or DvβGRP5.

**Figure S10**. Original, unaltered full blot images corresponding to Figure S4B. Input bait and prey samples and pull-down output fractions from GST pull-down assays assessing the association of GST-tagged DvHSP83 variants with His/V5-tagged PRRs. GST served as the bait control.

**Figure S11.** Original, unaltered full blot images corresponding to Figure S4C. Input and fungus-bound output fractions of HSP83-1Mut, HSP83-2Mut, and HSP83-3Mut in binding assays with PBS, Lp, L&O, or Om.

**Figure S12.** Original, unaltered full blot images corresponding to the βGRP3 assays in Figure S4D. Input and fungus-bound output fractions following pre-mixing of DvβGRP3 with HSP83-1Mut, HSP83-2Mut, or HSP83-3Mut.

**Figure S13.** Original, unaltered full blot images corresponding to the βGRP5 assays in Figure S4D. Input and fungus-bound output fractions following pre-mixing of DvβGRP5 with HSP83-1Mut, HSP83-2Mut, or HSP83-3Mut.

**Figure S14.** Original, unaltered full blot images corresponding to the PGRP-SA assays in Figure S4D. Input and fungus-bound output fractions following pre-mixing of DvPGRP-SA with HSP83-1Mut, HSP83-2Mut, or HSP83-3Mut.

3. Supplementary Tables S1–S5

**Table S1.** Primers used in this study.

| **Primer name** | **Direction** | **Sequence** |
| --- | --- | --- |
| q-Dva-Att-F | Forward | ACGGACTTCAAGGTGCAATC |
| q-Dva-Att-R | Reverse | ATGCGTATCCTGAACCATCC |
| q-Dva-Def1-F | Forward | GTTAAAGTGAACGATTCCACA |
| q-Dva-Def1-R | Reverse | GCGACACACGCAAACTTGGT |
| q-Dva-Def2-F | Forward | GTTAAAGTGAACGATTCCACT |
| q-Dva-Def2-R | Reverse | GCGACACACGCAAACTTGGT |
| q-Dva-Dpt-F | Forward | TCTACCCTGGACGATTCTCG |
| q-Dva-Dpt-R | Reverse | CCACTTTGTCTTTGCCCACT |
| q-Dva-βGRP3-F | Forward | TGTGGGCATTGAGAATCAGA |
| q-Dva-βGRP3-R | Reverse | TCCGGGTATTCTCCAAACAG |
| q-Dva-βGRP5-F | Forward | CCGACTCCCTCTATGGTGAA |
| q-Dva-βGRP5-R | Reverse | CCGTTTGGTAGCATTCTGGT |
| q-Dva-PGRP-SA-F | Forward | GTTCTAGCCTCAGCCAATGC |
| q-Dva-PGRP-SA-R | Reverse | CCGCCAATCATGAAGTTGTA |
| q-Dva-PGRP-SC2-F | Forward | TGGGAGATGGGAGTAGATCG |
| q-Dva-PGRP-SC2-R | Reverse | GATGGAGGCAAGTGATGGTT |
| q-Dva-βGRP1-F | Forward | TGCTAGAATTCGCACATTGG |
| q-Dva-βGRP1-R | Reverse | TTGGCCAGCTTCCATATTTC |
| q-Dva-βGRP2-F | Forward | GATATTGGCGGCAAACAAGT |
| q-Dva-βGRP2-R | Reverse | GCCATTTATTCCCAATGTGC |
| q-Dva-βGRP4-F | Forward | ACTGCCGAAACTGAGGACAC |
| q-Dva-βGRP4-R | Reverse | ATGGTACTTTGGCACGGAAG |
| q-Dva-Dorsal-L | Forward | CAAGCTGGCTGAGGATGTGA |
| q-Dva-Dorsal-R | Reverse | CTCATCCAACTCCGCCACTT |
| q-Dva-PGRP-SC1-F | Forward | TGCCAGTTGGTCTGGTTGTA |
| q-Dva-PGRP-SC1-R | Reverse | TCTGGACTAGAAGGGCGAGA |
| q-Dva-Serpin3-F | Forward | ATGCGCGATTAATCTTCTGG |
| q-Dva-Serpin3-R | Reverse | GGATCGCTGGTTAGTTCGAC |
| q-Dva-Serpin9-F | Forward | GTGGCTCTTTCGCCACTTAC |
| q-Dva-Serpin9-R | Reverse | TTCCGCTCAGTCTTTGGAAT |
| q-Dva-Serpin10-F | Forward | ATCGATGTGGATGAGGAAGG |
| q-Dva-Serpin10-R | Reverse | AGGGATGAGTGGCATTGAAC |
| q-Dva-HSP83-F | Forward | TTCCAGGCTGAAATCGCTCA |
| q-Dva-HSP83-R | Reverse | ACTTGGATGGGTTGGTGAGG |
| q-Dme-Def-L | Forward | TTCGTTCTCGTGGCTATCGC |
| q-Dme-Def-R | Reverse | TCCTCATGCACCAGGACATG |
| q-Dme-DptB-L | Forward | AACCACTGGCATATGCTCCC |
| q-Dme-DptB-R | Reverse | TCGAATCCTTGCTTTGGGCT |
| T7-dsHSP83-F | Forward | TAATACGACTCACTATAGGG ATCCCTCACCAACCCATCCA |
| T7-dsHSP83-R | Reverse | TAATACGACTCACTATAGGG GGCCTCATCATCGCTCAACT |
| T7-dsEGFP-F | Forward | TAATACGACTCACTATAGGG GGCAAGCTGACCCTGAAGTT |
| T7-dsEGFP-R | Reverse | TAATACGACTCACTATAGGG GTCCATGCCGAGAGTGATCC |

| ***Continued*** |  |  |
| --- | --- | --- |
| **Primer name** | **Direction** | **Sequence** |
| q-Dme-AttA-L | Forward | AGCAACTTCCCAGGAATCGG |
| q-Dme-AttA-R | Reverse | CGATCCCGTGAGATCCAAGG |
| q-Dme-AttB-L | Forward | CTTCGAGTTCCAGCGCAATG |
| q-Dme-AttB-R | Reverse | CCGATTCCTGGGAAGTTGCT |
| q-Dme-AttD-L | Forward | CAATGCTCGAGCCGGAGTAT |
| q-Dme-AttD-R | Reverse | GGAAGAGATTGGCTTGGGCT |
| q-Dme-Mtk-L | Forward | GCTACATCAGTGCTGGCAGA |
| q-Dme-Mtk-R | Reverse | ATTGGACCCGGTCTTGGTTG |
| q-Dva-rpL39-L | Forward | CTGCCCACAAGACGTTTA |
| q-Dva-rpL39-R | Reverse | GCCTCTTAGCGTTGTACCTA |
| q-Dme-rpL32-L | Forward | TACAGGCCCAAGATCGTGAAG |
| q-Dme-rpL32-R | Reverse | GACGCACTCTGTTGTCGATACC |

**Table S2.** Confidence metrics for exploratory AlphaFold Server models used to prioritize DvHSP83 residues for mutagenesis.

| **Predicted complex** | **ipTM** | **pTM** | **Role in prioritizing candidate residues** |
| --- | --- | --- | --- |
| 2×DvHSP83–DvβGRP3 | 0.43 | 0.52 | Contributed to E15, E61, and K64 prioritization |
| 2×DvHSP83–DvβGRP5 | 0.41 | 0.51 | Contributed to E15, E61, and K64 prioritization |
| 2×DvHSP83–DvPGRP-SA | 0.59 | 0.68 | Contributed to E15 prioritization |
| 2×DvHSP83–DvPGRP-SC2 | 0.54 | 0.65 | Nonbinding specificity comparator |

Each input comprised two full-length DvHSP83 molecules and one PRR, and only the top-ranked model returned by AlphaFold Server was analyzed. Candidate DvHSP83 residues were prioritized through screening based on static distances and manual comparison across the four models. E15 recurred in the DvPGRP-SA, DvβGRP3, and DvβGRP5 models, whereas E61 and K64 recurred in the DvβGRP3 and DvβGRP5 models. The ipTM values indicated limited confidence in the precise relative arrangement of the modeled proteins. Accordingly, the models were used only for exploratory residue prioritization and were not interpreted as experimentally resolved atomic interfaces.

**Table S3.** Peptide concentrations and total peptide amounts for each DIA sample.

| **Sample name** | **Concentration (μg/μL)** | **Sample volume (μL)** | **Total peptide amount (μg)** |
| --- | --- | --- | --- |
| iGFP+L&O1 | 0.89 | 19 | 16.91 |
| iGFP+L&O2 | 0.89 | 19 | 16.91 |
| iGFP+L&O3 | 0.92 | 19 | 17.48 |
| iGFP+PBS1 | 0.83 | 19 | 15.77 |
| iGFP+PBS2 | 0.89 | 19 | 16.91 |
| iGFP+PBS3 | 2.9 | 19 | 55.1 |
| iHSP83+L&O1 | 0.95 | 19 | 18.05 |
| iHSP83+L&O2 | 0.93 | 19 | 17.67 |
| iHSP83+L&O3 | 1 | 19 | 19 |
| iHSP83+PBS1 | 0.69 | 19 | 13.11 |
| iHSP83+PBS2 | 0.94 | 19 | 17.86 |
| iHSP83+PBS3 | 1.23 | 19 | 23.37 |
| L&O1 | 1.08 | 19 | 20.52 |
| L&O2 | 0.96 | 19 | 18.24 |
| L&O3 | 1.03 | 19 | 19.57 |
| Lp1 | 0.7 | 19 | 13.3 |
| Lp2 | 0.82 | 19 | 15.58 |
| Lp3 | 0.75 | 19 | 14.25 |
| Om1 | 0.58 | 19 | 11.02 |
| Om2 | 0.6 | 19 | 11.4 |
| Om3 | 0.9 | 19 | 17.1 |
| PBS1 | 0.85 | 19 | 16.15 |
| PBS2 | 0.77 | 19 | 14.63 |
| PBS3 | 0.88 | 19 | 16.72 |

**Table S4.** Descriptions of enriched KEGG terms shown in Figure 3C.

| **KEGG term ID** | ***P* value** | **Description** |
| --- | --- | --- |
| tca00350 | 1.33E-05 | Tyrosine metabolism |
| tca00790 | 5.46E-05 | Folate biosynthesis |
| tca00380 | 0.008567469 | Tryptophan metabolism |
| tca00360 | 0.014206882 | Phenylalanine metabolism |
| tca04624 | 0.020527293 | Toll and IMD signaling pathway |
| tca02010 | 0.026255423 | ABC transporters |

**Table S5.** Key Resources Table.

| **REAGENT or RESOURCE** | **SOURCE** | **IDENTIFIER** |
| --- | --- | --- |
| **Biological samples** |  |  |
| Red turpentine beetle (*Dendroctonus valens*) | Field collected | N/A |
| Chinese pine (*Pinus tabuliformis*) | Field site | N/A |
| **Fungal and bacterial strains** |  |  |
| *Leptographium procerum* | Lu et al.^[33]^ | CMW 25624 |
| *Ophiostoma minus* | Lu et al.^[33]^ | CMW 26254 |
| *Escherichia coli* DH5α | TIANGEN | Cat#CB101 |
| *Escherichia coli* BL21(DE3) | TIANGEN | Cat#CB105 |
| **Experimental models: Cell lines** |  |  |
| *Drosophila* S2 cell | Gibco | R69007 |
| **Recombinant DNA** |  |  |
| pET-28a-DvPGRP-SA | this paper | N/A |
| pET-28a-DvPGRP-SC2 | this paper | N/A |
| pET-28a-DvβGRP3 | this paper | N/A |
| pET-28a-DvβGRP5 | this paper | N/A |
| pET-28a-DvDorsal | this paper | N/A |
| pET-28a-DvRelish2 | this paper | N/A |
| pET-28a-DvHSP83 | this paper | N/A |
| pET-28a-DvHSP83-E15A | this paper | N/A |
| pET-28a-DvHSP83-E61A/K64A | this paper | N/A |
| pET-28a-DvHSP83-E15A/E61A/K64A | this paper | N/A |
| pGEX-4T-1-DvHSP83 | this paper | N/A |
| pGEX-4T-1-DvHSP83-E15A | this paper | N/A |
| pGEX-4T-1-DvHSP83-E61A/K64A | this paper | N/A |
| pGEX-4T-1-DvHSP83-E15A/E61A/K64A | this paper | N/A |
| pAc5.1b-DvHSP83-V5 | this paper | N/A |
| pAc5.1b-DvDorsal-V5 | this paper | N/A |
| **Antibodies** |  |  |
| Rabbit polyclonal anti-PGRP-SA | this paper | N/A |
| Rabbit polyclonal anti-PGRP-SC2 | this paper | N/A |
| Rabbit polyclonal anti-βGRP3 | this paper | N/A |
| Rabbit polyclonal anti-βGRP5 | this paper | N/A |
| Rabbit polyclonal anti-Dorsal | this paper | N/A |
| Rabbit polyclonal anti-HSP83 | this paper | N/A |
| Mouse monoclonal anti-Hsp90 | Abcam | ab59459 |
| Rabbit monoclonal anti-β-Tubulin | Absin | abs171597 |
| Mouse monoclonal anti-GST | EASYBIO | BE2065 |
| Mouse monoclonal anti-V5 | Invitrogen | R960-25 |
| Goat Anti-Rabbit IgG | EASYBIO | BE0101 |
| Goat anti-Mouse IgG | EASYBIO | BE0102 |
| Goat Anti-Mouse IgG (H+L) Alexa Fluor 594 | Abcam | ab150116 |
| Donkey Anti-Rabbit IgG (H+L) Alexa Fluor 647 | Abcam | ab150075 |
| Goat Anti-Rabbit IgG (H+L) Alexa Fluor 594 | Abcam | ab150080 |

| ***Continued*** |  |  |
| --- | --- | --- |
| **REAGENT or RESOURCE** | **SOURCE** | **IDENTIFIER** |
| **Oligonucleotides** |  |  |
| Primers for qPCR and RNAi, see Table S1 | this paper | N/A |
| **Deposited Data** |  |  |
| RNA-seq raw data | This paper | SRA: PRJNA1178106 |
| Proteomics data | This paper | ProteomeXchange: PXD069324 |
| **Chemicals, peptides, and recombinant proteins** |  |  |
| GeneJET RNA Purification Kit | Thermo Scientific | Cat#K0731 |
| PrimeScript RT reagent Kit with gDNA Eraser | Takara | Cat#RR047A |
| TB Green Premix Ex Taq II | Takara | Cat#RR820A |
| RIPA Lysis Buffer (Strong) | Cwbio | Cat#CW2333S |
| RIPA Lysis Buffer (Weak) | Beyotime | Cat#P0013D |
| Protease Inhibitor Cocktail (10×) | Thermo Scientific | Cat#87786 |
| Protease Inhibitor Cocktail | Beijing Solarbio Science & Technology Co., Ltd. | Cat#P6735 |
| BCA Protein Assay Kit | Cwbio | Cat#CW0014S |
| StartingBlock T20 (TBS) Blocking Buffer | Thermo Scientific | Cat#37543 |
| SuperSignal West Plus Chemiluminescent Substrate | Thermo Scientific | Cat#34580 |
| Wheat Germ Agglutinin, Alexa Fluor 488 Conjugate | Thermo Fisher Scientific | Cat#W11261 |
| DAPI | Macklin | Cat#D807959 |
| Cellfectin II Reagent | Gibco | Cat#10362100 |
| **Software and Algorithms** |  |  |
| GraphPad Prism 10 | GraphPad | https://www.graphpad.com/ |
| R 4.4.0 | The R Project | https://www.r-project.org/ |
| Cytoscape 3.10.2 | Cytoscape | https://cytoscape.org/ |
| Online bioinformatics platform | Tang et al.^[34]^ | http://www.bioinformatics.com.cn |
| MAFFT v7.526 | Katoh et al.^[25]^ | https://mafft.cbrc.jp/alignment/software/ |
| IQ-TREE v2.3.6 | Kalyaanamoorthy et al.^[26]^ | https://github.com/iqtree/iqtree2 |
| ESPript 3.0 Server | Robert et al.^[31]^ | https://espript.ibcp.fr/ESPript/ESPript/ |
| AlphaFold Server (powered by AlphaFold 3) | Google DeepMind | https://alphafoldserver.com/ |
| PyMOL v3.0.3 | Schrödinger, LLC | https://www.pymol.org/ |

4. Supplementary References

1. Z. Liu, B. Wang, B. Xu, J. Sun, “Monoterpene variation mediated attack preference evolution of the bark beetle *Dendroctonus valens*,” *PLOS ONE* 6, no. 7 (2011): e22005. <https://doi.org/10.1371/journal.pone.0022005>.

2. Z. Liu, L. Xing, W. Huang, B. Liu, F. Wan, K. F. Raffa, R. W. Hofstetter, W. Qian, J. Sun, “Chromosome-level genome assembly and population genomic analyses provide insights into adaptive evolution of the red turpentine beetle, *Dendroctonus valens*,” *BMC Biology* 20, no. 1 (2022): 190. <https://doi.org/10.1186/s12915-022-01388-y>.

3. L. Xu, Y. Zhang, S. Zhang, J. Deng, M. Lu, L. Zhang, J. Zhang, “Comparative analysis of the immune system of an invasive bark beetle, *Dendroctonus valens*, infected by an entomopathogenic fungus,” *Developmental and Comparative Immunology* 88 (2018): 65. <https://doi.org/10.1016/j.dci.2018.07.002>.

4. F. Liu, F. Ye, C. Cheng, Z. Kang, H. Kou, J. Sun, “Symbiotic microbes aid host adaptation by metabolizing a deterrent host pine carbohydrate d-pinitol in a beetle-fungus invasive complex,” *Science Advances* 8, no. 51 (2022): eadd5051. <https://doi.org/10.1126/sciadv.add5051>.

5. Y. H. Wang, Y. Hu, L. S. Xing, H. Jiang, S. N. Hu, A. S. Raikhel, Z. Zou, “A Critical Role for CLSP2 in the Modulation of Antifungal Immune Response in Mosquitoes,” *PLOS Pathogens* 11, no. 6 (2015): e1004931. <https://doi.org/10.1371/journal.ppat.1004931>.

6. C. Yuan, Y. Sun, J. Chen, Q. Xu, X. Zhou, Z. Zou, Q. Xia, “*Haemaphysalis longicornis* subolesin controls the infection and transmission of severe fever with thrombocytopenia syndrome virus,” *NPJ Vaccines* 10, no. 1 (2025): 17. <https://doi.org/10.1038/s41541-024-01061-1>.

7. Y. Wang, H. Jiang, Y. Cheng, C. An, Y. Chu, A. S. Raikhel, Z. Zou, “Activation of *Aedes aegypti* prophenoloxidase-3 and its role in the immune response against entomopathogenic fungi,” *Insect Molecular Biology* 26, no. 5 (2017): 552–563. <https://doi.org/10.1111/imb.12318>.

8. T. Lu, Y. Ji, M. Chang, X. Zhang, Y. Wang, Z. Zou, “The accumulation of modular serine protease mediated by a novel circRNA sponging miRNA increases *Aedes aegypti* immunity to fungus,” *BMC Biology* 22, no. 1 (2024): 7. <https://doi.org/10.1186/s12915-024-01811-6>.

9. N. Liu, Y. Xu, Q. Li, Y. Cao, D. Yang, S. Liu, X. Wang, Y. Mi, Y. Liu, C. Ding, Y. Liu, Y. Li, Y. W. Yuan, G. Gao, J. Chen, W. Qian, X. Zhang, “A lncRNA fine-tunes salicylic acid biosynthesis to balance plant immunity and growth,” *Cell Host & Microbe* 30, no. 8 (2022): 1124. <https://doi.org/10.1016/j.chom.2022.07.001>.

10. J. Abramson, J. Adler, J. Dunger, R. Evans, T. Green, A. Pritzel, O. Ronneberger, L. Willmore, A. J. Ballard, J. Bambrick, S. W. Bodenstein, D. A. Evans, C. C. Hung, M. O'Neill, D. Reiman, K. Tunyasuvunakool, Z. Wu, A. Žemgulytė, E. Arvaniti, C. Beattie, O. Bertolli, A. Bridgland, A. Cherepanov, M. Congreve, A. I. Cowen-Rivers, A. Cowie, M. Figurnov, F. B. Fuchs, H. Gladman, R. Jain, Y. A. Khan, C. M. R. Low, K. Perlin, A. Potapenko, P. Savy, S. Singh, A. Stecula, A. Thillaisundaram, C. Tong, S. Yakneen, E. D. Zhong, M. Zielinski, A. Žídek, V. Bapst, P. Kohli, M. Jaderberg, D. Hassabis, J. M. Jumper, “Accurate structure prediction of biomolecular interactions with AlphaFold 3,” *Nature* 630 (2024): 493–500. <https://doi.org/10.1038/s41586-024-07487-w>.

11. Q. T. Yang, L. L. Zhao, Z. Zou, Y. H. Wang, R. Y. Ma, “Peptidoglycan recognition protein AaPGRP-LC is involved in immune responses against bacteria in *Aedes aegypti* (Diptera: Culicidae),” *Acta Entomologica Sinica* 61, no. 1 (2018): 25. <https://doi.org/10.16380/j.kcxb.2018.01.004>.

12. Q. Meng, P. P. Wu, M. M. Li, R. H. Shu, G. L. Zhou, J. H. Zhang, H. Zhang, H. Jiang, Q. L. Qin, Z. Zou, “Distinct Responses of *Thitarodes xiaojinensis* β-1,3-Glucan Recognition Protein-1 and Immulectin-8 to *Ophiocordyceps sinensis* and *Cordyceps militaris* Infection,” *The Journal of Immunology* 207, no. 1 (2021): 200. <https://doi.org/10.4049/jimmunol.2000447>.

13. M. M. Chang, Y. H. Wang, Q. T. Yang, X. L. Wang, M. Wang, A. S. Raikhel, Z. Zou, “Regulation of antimicrobial peptides by juvenile hormone and its receptor, Methoprene-tolerant, in the mosquito *Aedes aegypti*,” *Insect Biochemistry and Molecular Biology* 128 (2021): 103509. <https://doi.org/10.1016/j.ibmb.2020.103509>.

14. Z. Lin, Y. Cheng, R. J. Wang, J. Du, O. Volovych, J. C. Li, Y. Hu, Z. Y. Lu, Z. Lu, Z. Zou, “A Metalloprotease Homolog Venom Protein From a Parasitoid Wasp Suppresses the Toll Pathway in Host Hemocytes,” *Frontiers in Immunology* 9 (2018): 2301. <https://doi.org/10.3389/fimmu.2018.02301>.

15. C. Yuan, L. Xing, M. Wang, X. Wang, M. Yin, Q. Wang, Z. Hu, Z. Zou, “Inhibition of melanization by serpin-5 and serpin-9 promotes baculovirus infection in cotton bollworm *Helicoverpa armigera*,” *PLOS Pathogens* 13, no. 9 (2017): e1006645. <https://doi.org/10.1371/journal.ppat.1006645>.

16. X. Sun, X. Wang, K. Shi, X. Lyu, J. Sun, A. S. Raikhel, Z. Zou, “Leucine aminopeptidase1 controls egg deposition and hatchability in male *Aedes aegypti* mosquitoes,” *Nature Communications* 15, no. 1 (2024): 106. <https://doi.org/10.1038/s41467-023-44444-z>.

17. L. C. Gillet, P. Navarro, S. Tate, H. Röst, N. Selevsek, L. Reiter, R. Bonner, R. Aebersold, “Targeted data extraction of the MS/MS spectra generated by data-independent acquisition: a new concept for consistent and accurate proteome analysis,” *Molecular & Cellular Proteomics* 11, no. 6 (2012): O111.016717. <https://doi.org/10.1074/mcp.O111.016717>.

18. E. W. Deutsch, N. Bandeira, Y. Perez-Riverol, V. Sharma, J. J. Carver, L. Mendoza, D. J. Kundu, S. Wang, C. Bandla, S. Kamatchinathan, S. Hewapathirana, B. S. Pullman, J. Wertz, Z. Sun, S. Kawano, S. Okuda, Y. Watanabe, B. MacLean, M. J. MacCoss, Y. Zhu, Y. Ishihama, J. A. Vizcaíno, “The ProteomeXchange consortium at 10 years: 2023 update,” *Nucleic Acids Research* 51, no. D1 (2023): D1539. <https://doi.org/10.1093/nar/gkac1040>.

19. Z. Zou, S. W. Shin, K. S. Alvarez, G. Bian, V. Kokoza, A. S. Raikhel, “Mosquito RUNX4 in the immune regulation of PPO gene expression and its effect on avian malaria parasite infection,” *Proceedings of the National Academy of Sciences of the United States of America* 105, no. 47 (2008): 18454. <https://doi.org/10.1073/pnas.0804658105>.

20. M. Wang, Y. Wang, M. Chang, X. Wang, Z. Shi, A. S. Raikhel, Z. Zou, “Ecdysone signaling mediates the trade-off between immunity and reproduction via suppression of amyloids in the mosquito *Aedes aegypti*,” *PLOS Pathogens* 18, no. 9 (2022): e1010837. <https://doi.org/10.1371/journal.ppat.1010837>.

21. A. Öztürk-Çolak, S. J. Marygold, G. Antonazzo, H. Attrill, D. Goutte-Gattat, V. K. Jenkins, B. B. Matthews, G. Millburn, G. Dos Santos, C. J. Tabone, “FlyBase: updates to the *Drosophila* genes and genomes database,” *Genetics* 227, no. 1 (2024): iyad211. <https://doi.org/10.1093/genetics/iyad211>.

22. Y. Kawahara, M. de la Bastide, J. P. Hamilton, H. Kanamori, W. R. McCombie, S. Ouyang, D. C. Schwartz, T. Tanaka, J. Wu, S. Zhou, K. L. Childs, R. M. Davidson, H. Lin, L. Quesada-Ocampo, B. Vaillancourt, H. Sakai, S. S. Lee, J. Kim, H. Numa, T. Itoh, C. R. Buell, T. Matsumoto, “Improvement of the *Oryza sativa* Nipponbare reference genome using next generation sequence and optical map data,” *Rice* 6, no. 1 (2013): 4. <https://doi.org/10.1186/1939-8433-6-4>.

23. J. P. Hamilton, C. Li, C. R. Buell, “The rice genome annotation project: an updated database for mining the rice genome,” *Nucleic Acids Research* 53, no. D1 (2025): D1614. <https://doi.org/10.1093/nar/gkae1061>.

24. P. Lamesch, T. Z. Berardini, D. Li, D. Swarbreck, C. Wilks, R. Sasidharan, R. Muller, K. Dreher, D. L. Alexander, M. Garcia-Hernandez, A. S. Karthikeyan, C. H. Lee, W. D. Nelson, L. Ploetz, S. Singh, A. Wensel, E. Huala, “The *Arabidopsis* Information Resource (TAIR): improved gene annotation and new tools,” *Nucleic Acids Research* 40 (2012): D1202. <https://doi.org/10.1093/nar/gkr1090>.

25. K. Katoh, D. M. Standley, “MAFFT multiple sequence alignment software version 7: improvements in performance and usability,” *Molecular Biology and Evolution* 30, no. 4 (2013): 772. <https://doi.org/10.1093/molbev/mst010>.

26. S. Kalyaanamoorthy, B. Q. Minh, T. K. F. Wong, A. von Haeseler, L. S. Jermiin, “ModelFinder: fast model selection for accurate phylogenetic estimates,” *Nature Methods* 14, no. 6 (2017): 587. <https://doi.org/10.1038/nmeth.4285>.

27. B. Q. Minh, H. A. Schmidt, O. Chernomor, D. Schrempf, M. D. Woodhams, A. von Haeseler, R. Lanfear, “IQ-TREE 2: New Models and Efficient Methods for Phylogenetic Inference in the Genomic Era,” *Molecular Biology and Evolution* 37, no. 5 (2020): 1530. <https://doi.org/10.1093/molbev/msaa015>.

28. A. Marchler-Bauer, S. H. Bryant, “CD-Search: protein domain annotations on the fly,” *Nucleic Acids Research* 32 (2004): W327. <https://doi.org/10.1093/nar/gkh454>.

29. A. Marchler-Bauer, S. Lu, J. B. Anderson, F. Chitsaz, M. K. Derbyshire, C. DeWeese-Scott, J. H. Fong, L. Y. Geer, R. C. Geer, N. R. Gonzales, M. Gwadz, D. I. Hurwitz, J. D. Jackson, Z. Ke, C. J. Lanczycki, F. Lu, G. H. Marchler, M. Mullokandov, M. V. Omelchenko, C. L. Robertson, J. S. Song, N. Thanki, R. A. Yamashita, D. Zhang, N. Zhang, C. Zheng, S. H. Bryant, “CDD: a Conserved Domain Database for the functional annotation of proteins,” *Nucleic Acids Research* 39 (2011): D225. <https://doi.org/10.1093/nar/gkq1189>.

30. E. Gasteiger, C. Hoogland, A. Gattiker, S. Duvaud, M. R. Wilkins, R. D. Appel, A. Bairoch, in *The Proteomics Protocols Handbook* (Ed.: J. M. Walker), Humana Press, Totowa, NJ (2005).

31. X. Robert, P. Gouet, “Deciphering key features in protein structures with the new ENDscript server,” *Nucleic Acids Research* 42 (2014): W320. <https://doi.org/10.1093/nar/gku316>.

32. G. Zhang, Y. Li, D. Huang, L. Tan, W. Li, H. Peng, Y. Ye, L. Jiang, J. Xie, J. Zhang, S. Cui, “Identification of cellular senescence-associated hub genes and molecular targeting mechanisms of thalidomide in lupus nephritis (LN),” *Autoimmunity* 58, no. 1 (2025): 2561619. <https://doi.org/10.1080/08916934.2025.2561619>.

33. M. Lu, X. D. Zhou, Z. W. De Beer, M. J. Wingfield, J. H. Sun, “Ophiostomatoid fungi associated with the invasive pine-infesting bark beetle, *Dendroctonus valens*, in China,” *Fungal Diversity* 38 (2009): 133.

34. D. Tang, M. Chen, X. Huang, G. Zhang, L. Zeng, G. Zhang, S. Wu, Y. Wang, “SRplot: A free online platform for data visualization and graphing,” *PLOS ONE* 18, no. 11 (2023): e0294236. <https://doi.org/10.1371/journal.pone.0294236>.
